# Supplementary material for: The Pro-apoptotic STK38 Kinase Is a New Beclin1 Partner Positively Regulating Autophagy
Source: Curr Biol. 2015 Oct 5;25(19):2479–92. doi: 10.1016/j.cub.2015.08.031 (PMC4598746; doi:10.1016/j.cub.2015.08.031)
Supplement: Document S1. Supplemental Discussion, Supplemental Experimental Procedures, and Figures S1–S7 [file mmc1.pdf]

Current Biology

Supplemental Information

The Pro-apoptotic STK38 Kinase Is a New Beclin1 Partner Positively Regulating Autophagy

Carine Joffre, Nicolas Dupont, Lily Hoa, Valenti Gomez, Raul Pardo, Catarina Gonçalves-Pimentel, Pauline Achard, Audrey Bettoun, Brigitte Meunier, Chantal Bauvy, Ilaria Cascone, Patrice Codogno, Manolis Fanto, Alexander Hergovich, and Jacques Camonis

Figure S1

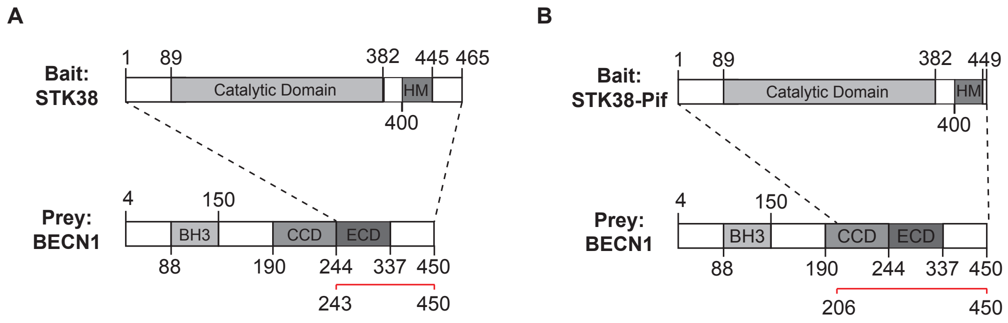

Yeast two hybrid interactions between STK38/STK38-PIF and Beclin1 (in support of Figure 1).

Primary structure of human STK38 (**A**), STK-PIF (**B**) and Beclin1 illustrating yeast two-hybrid results. Domains required for interactions between STK38 and Beclin1 are indicated. CCD: coiled-coil domain; ECD: evolutionarily conserved domain, HM: hydrophobic motif.

Figure S2

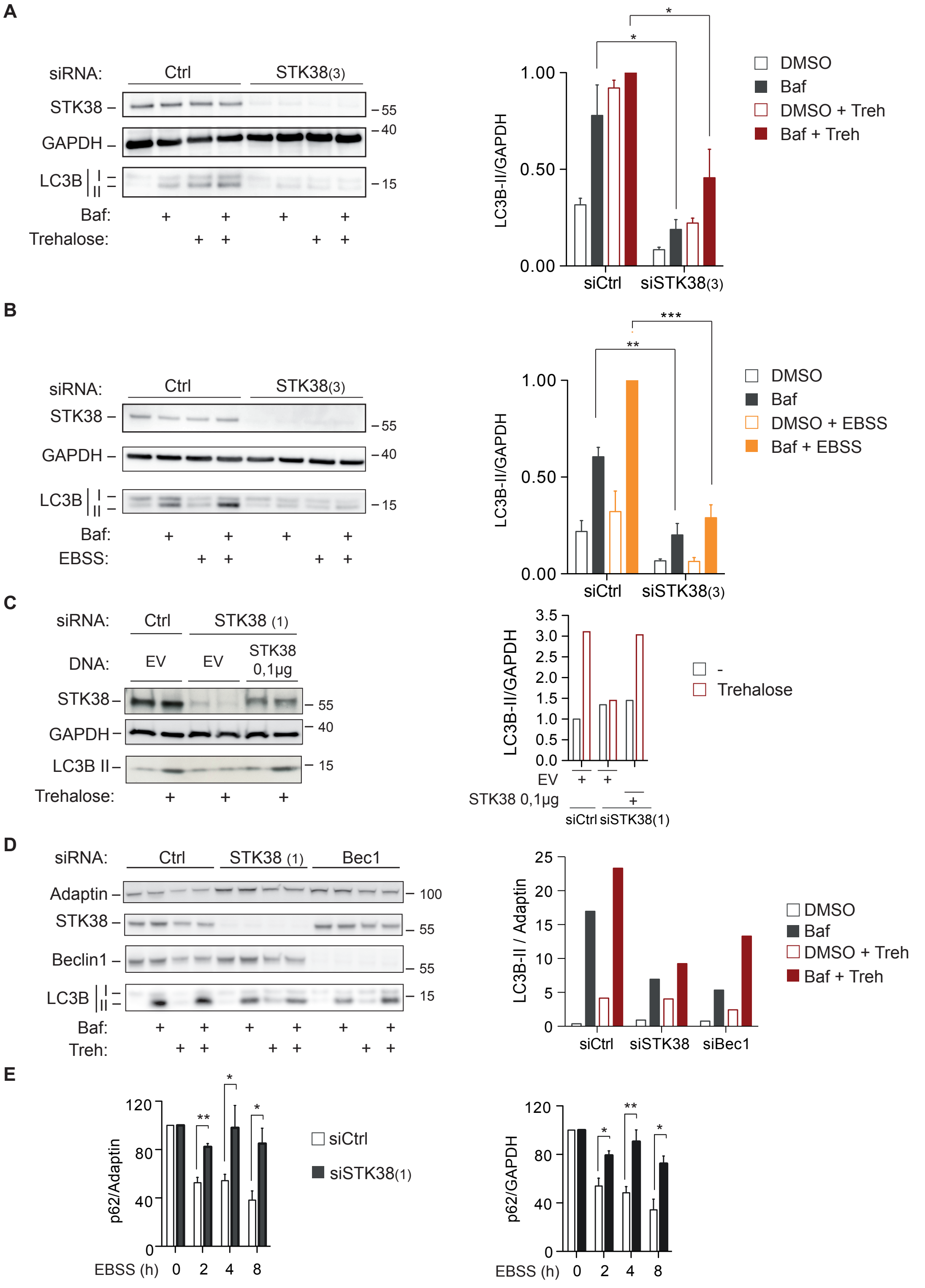
A
siRNA: Ctrl STK38(3)
STK38
GAPDH
LC3B I II
55
40
15
Baf:
Trehalose:
LC3B-II/GAPDH
1.00
0.50
0.00
siCtrl
siSTK38(3)
DMSO
Baf
DMSO + Treh
Baf + Treh
B
siRNA: Ctrl STK38(3)
STK38
GAPDH
LC3B I II
Baf:
EBSS:
LC3B-II/GAPDH
DMSO
Baf
DMSO + EBSS
Baf + EBSS
C
siRNA: Ctrl STK38 (1)
DNA: EV EV STK38 0,1µg
STK38
GAPDH
LC3B II
Trehalose:
LC3B-II/GAPDH
-
Trehalose
EV
STK38 0,1µg
siCtrl siSTK38(1)
D
siRNA: Ctrl STK38 (1) Bec1
Adaptin
STK38
Beclin1
LC3B I II
100
Baf:
Treh:
LC3B-II / Adaptin
siCtrl siSTK38 siBec1
DMSO
Baf
DMSO + Treh
Baf + Treh
E
p62/Adaptin
EBSS (h) 0 2 4 8
siCtrl
siSTK38(1)
p62/GAPDH
EBSS (h) 0 2 4 8

Data in support of Figure 2.

(A,B) STK38 depletion impairs the dynamics of LC3B-II levels upon autophagy induction. HeLa cells were transfected with indicated siRNAs. 72 h later, cells were incubated with Trehalose for 16 h (A) or EBSS for 4 h (B) with or without BafilomycinA1 (Baf) for 4 h, followed by immunoblotting using indicated antibodies (left panels). Histograms represent LC3B-II/GAPDH ratios obtained by densitometric analysis (n=3 +/- sem) of Western blots (right panels).

(C) RNAi-resistant STK38(wt) restores autophagy in STK38-depleted cells. STK38-depleted HeLa cells (48 h) were transfected with RNAi-resistant STK38 wild-type. 24 h later, cells were treated with Trehalose and LC3B-II levels were assessed by Western blots (left panel). Histogram shows LC3B-II/GAPDH ratios obtained by densitometric analysis of Western blots (right panel).

(D) STK38 is important for autophagy induction similar to Beclin1. HeLa cells were transfected with indicated siRNAs. 72 h later cells were treated with Trehalose for 16 h with or without Bafilomycin for 2 h, followed by processing for immunoblotting using indicated antibodies (left panel). Histograms show LC3B-II/Adaptin ratios obtained by densitometric analysis (right panel).

(E) STK38 depletion impairs the dynamics of p62 levels upon autophagy induction. HeLa cells were transfected with indicated siRNAs. 72 h later cells were subjected to EBSS treatment as indicated. p62 level was assessed by Western blotting (left panel). Histograms show p62/Adaptin and p62/GAPDH ratios obtained by densitometric analysis ($n \geq 3$ +/- sem) of Western blots (right panel). To determine statistically significant differences unpaired two-tailed Student's t-tests were carried out (* $p<0.05$, ** $p<0.01$, *** $p<0.001$).

Figure S3

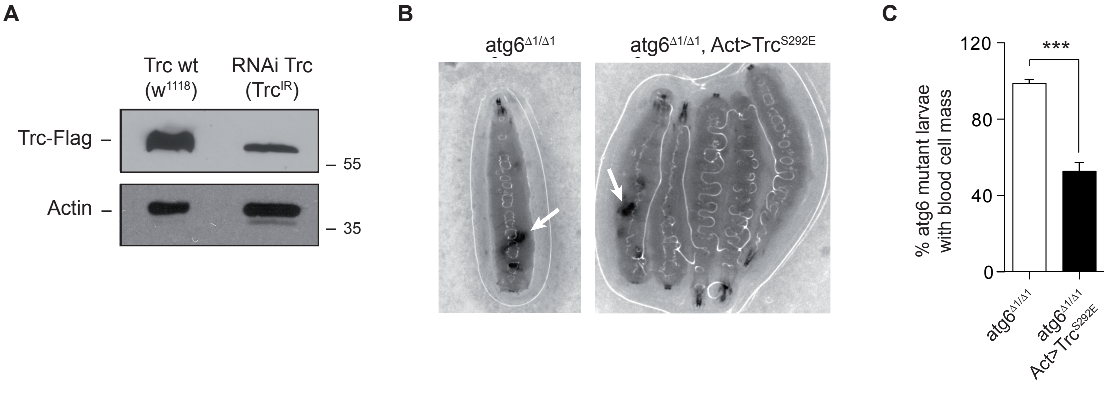

Data in support of Figure 3.

(A) Validation of Trc depletion by RNAi Trc. *Drosophila* heads with the indicated genotypes were processed for immunoblotting with the indicated antibodies. Noteworthy, exogenous Flag-tagged wild-type Trc was depleted in flies expressing the RNAi transgene directed against Trc.

(B,C) Activated Trc (fly STK38) can partially compensate for *atg6* loss-of-function. (B) Brightfield images of third instar fly larvae of an *atg6* mutant (left panel) and *atg6* mutants expressing activated Trc^{S292E} (right panel). Melanotic blood cell masses are visible as black masses (indicated by arrows). (C) Histograms show the quantification of larvae displaying blood cell masses (n=70). To determine statistically significant differences unpaired two-tailed Student's t-tests were carried out (*** $p<0.001$).

Figure S4

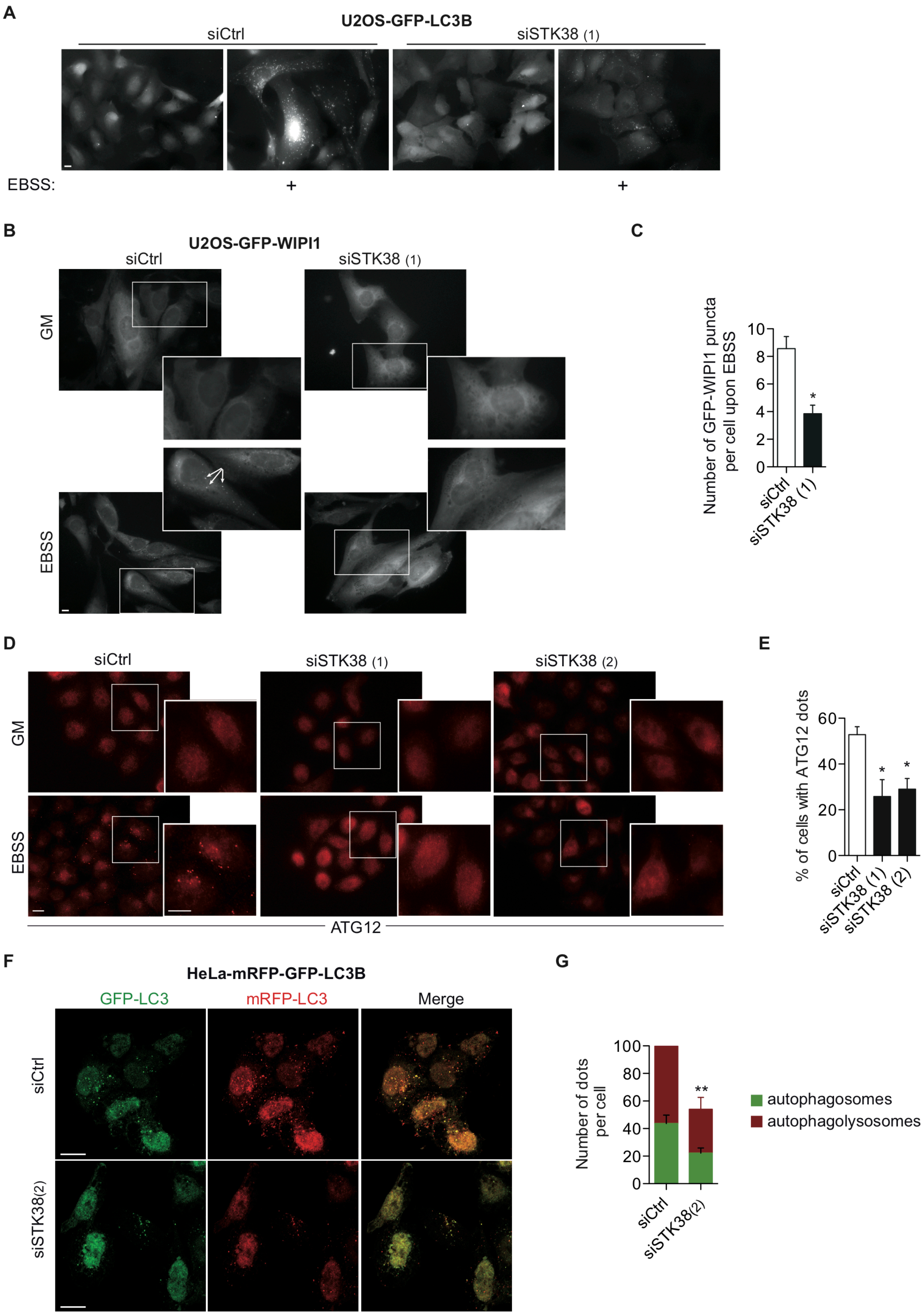

A
U2OS-GFP-LC3B
siCtrl
siSTK38 (1)
EBSS:
+
+
B
U2OS-GFP-WIPI1
siCtrl
siSTK38 (1)
GM
EBSS
C
Number of GFP-WIPI1 puncta per cell upon EBSS
10
8
6
4
2
0
*
siCtrl
siSTK38 (1)
D
siCtrl
siSTK38 (1)
siSTK38 (2)
GM
EBSS
ATG12
E
% of cells with ATG12 dots
60
40
20
0
*
*
siCtrl
siSTK38 (1)
siSTK38 (2)
F
HeLa-mRFP-GFP-LC3B
GFP-LC3
mRFP-LC3
Merge
siCtrl
siSTK38(2)
G
Number of dots per cell
100
80
60
40
20
0
**
siCtrl
siSTK38(2)
autophagosomes
autophagolysosomes

STK38 plays a role in early autophagic events (in support of Figure 4).

(A) U2OS cells stably expressing GFP-LC3B were transfected with indicated siRNAs. 72 h later cells were treated with EBSS for 2 h and fixed. Representative pictures are shown.

(B) U2OS cells stably expressing GFP-WIPI1 were transfected with indicated siRNAs. 72 h later cells were treated with EBSS for 2 h and fixed. Representative pictures are shown.

(C) Quantifications for experiments in (B). The histogram represents the number of GFP-WIPI1 puncta per cell upon EBSS treatment, quantifications performed with Image J software (n=3 +/- sem).

(D) HeLa cells were transfected with indicated siRNAs. 72 h later, cells were treated with EBSS for 2 h and stained for ATG12. Representative confocal pictures are shown. Scale bar: 10μM

(E) Quantification of experiments shown in (D). Histograms represent the percentage of cells displaying at least one ATG12 dot (n=3 +/- sem).

(F) HeLa-mRFP-GFP-LC3B cells were transfected with indicated siRNAs. 72 h after plating, cells were treated with EBSS for 2h and processed for immunofluorescence. Representative images are shown. Scale bar: 10 μM

(G) Quantification of experiments shown in (F). Histograms display the percentage of autophagosomes (yellow dots in F) and autophagolysosomes (red dots in F) per cell (n=3 +/- sem).

Figure S5

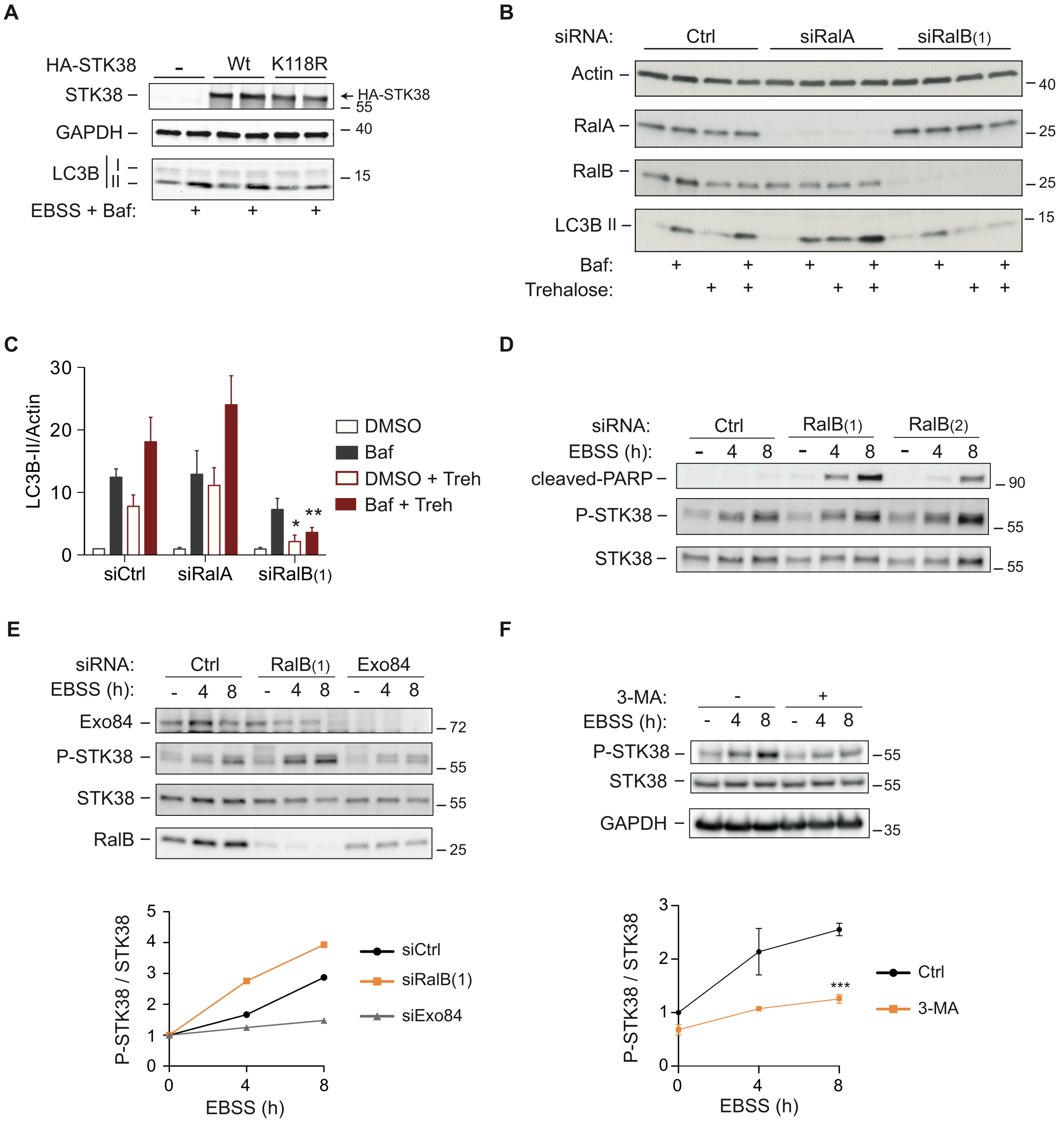

Data in support of Figure 6.

(A) Dominant negative kinase-dead STK38 interferes with autophagy induction. HeLa cells were transfected with an empty vector (-) or with a plasmid expressing either an HA tagged Wt STK38 or a kinase dead STK38 form. 24 h later cells were treated with EBSS for 2 h, followed by processing for immunoblotting using indicated antibodies. Wt, wild type; K118R, kinase-dead.

(B,C,D) RalB depletion blocks autophagy, but promotes apoptosis and STK38 activation. (B) HeLa cells were transfected with indicated siRNAs. 48 h later cells were treated with Trehalose for 16 h, followed by processing for immunoblotting using indicated antibodies. (C) Quantifications for experiments in (B). The histogram shows the LC3B-II/Actin ratios

obtained by densitometric analysis of Western blots (n=3 +/- sem). (D) HeLa cells were transfected with indicated siRNAs. 72 h later cells were treated with EBSS for the indicated times followed by processing for immunoblotting using indicated antibodies.

(E,F) Exo84 depletion and 3-MA treatment block EBSS-induced STK38 activation. (E) HeLa cells were transfected with indicated siRNAs. 72 h later cells were treated with EBSS for the indicated times followed by processing for immunoblotting using indicated antibodies. (F) HeLa cells were treated with 3-methyladenine (10mM) and stimulated with EBSS as indicated followed by processing for immunoblotting.

Figure S6

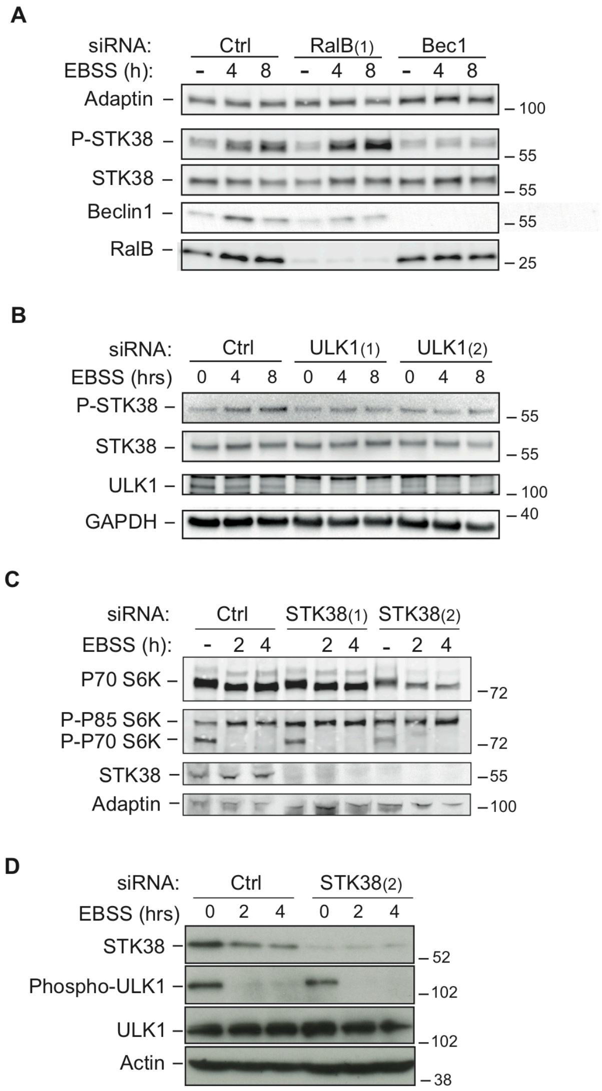

Beclin1 and ULK1 depletion impairs EBSS-induced STK38 activation, while STK38 depletion does not affect the suppression of mTOR-mediated phosphorylation of p70(S6K) on S389 and ULK1 on S757 upon nutrient deprivation (in support of Figure 6)
(A,B) HeLa cells were transfected with indicated siRNAs. 72 h later cells were treated with EBSS for the indicated times followed by processing for immunoblotting using indicated antibodies. Noteworthy, the EBSS-induced phosphorylation of STK38 is diminished in Beclin1 (A) and ULK1 (B) depleted cells.
(C,D) HeLa cells were transfected with indicated siRNAs. 72 h later cells were treated with EBSS for the indicated times followed by processing for immunoblotting using indicated antibodies. p70(S6K) and ULK1 phosphorylation by mTOR is decreased in control and STK38-depleted cells upon EBSS treatment, suggesting that STK38 is dispensable for the suppression of mTOR upon nutrient deprivation.

Figure S7

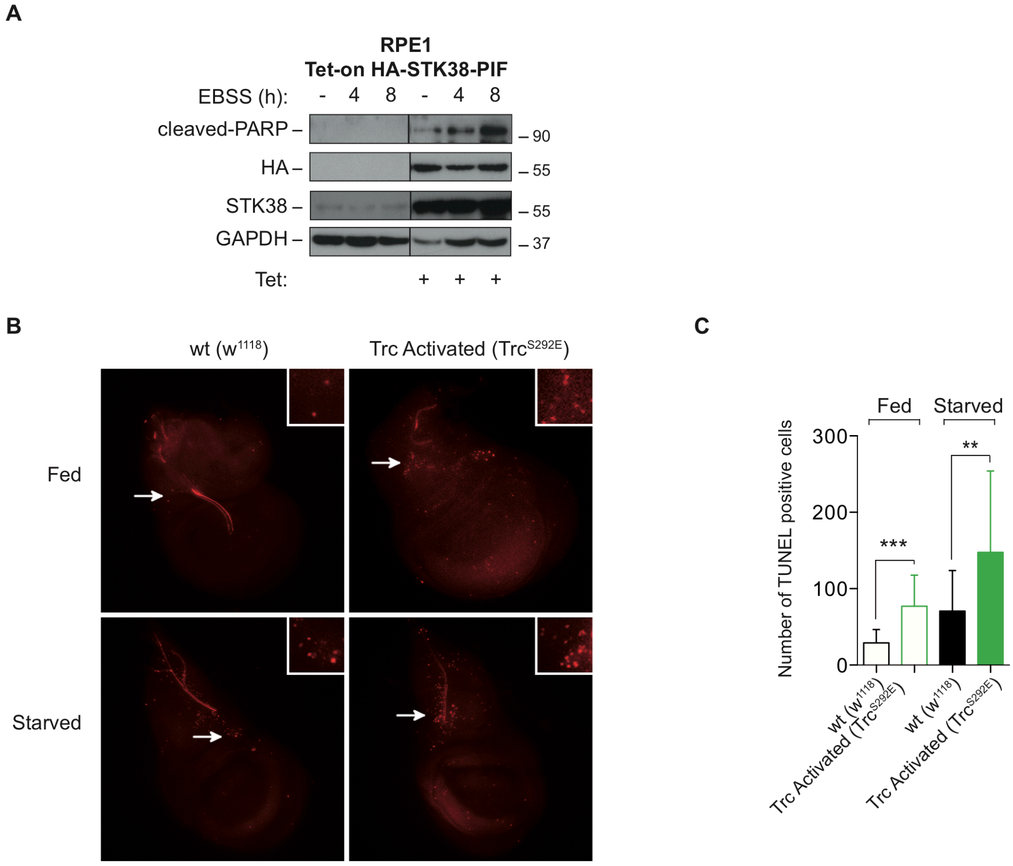

Data in support of Figure 7.

(A) Increased STK38 activity is sufficient to increase EBSS-induced apoptosis. RPE1 Tet-on HA-STK38-PIF cells were incubated with (+) or without tetracycline (2 µg/ml) for 24 h, before EBSS treatment as indicated and subsequent processing for immunoblotting using indicated antibodies. The Western blots shown were assembled from the same exposure.

(B,C) Activated Trc can drive increased apoptosis in fly larvae. (B) Fluorescent images of TUNEL staining of wing discs from fed or starved fly larvae expressing either no Gal4 driver (control Trc wt) or activated Trc^{S292E}. Magnifications of areas indicated by white arrows are shown as insets. Apoptotic cells are shown in red. (C) Histograms show the quantification of the number of TUNEL positive cells detected in each condition (n=20). To determine statistically significant differences unpaired two-tailed Student's t-tests were carried out (** $P<0.01$, *** $p<0.001$).

Supplemental Discussion

As a protein kinase [S1], STK38 represents a potentially interesting drug target to modulate autophagy activity in human diseases associated with deregulated autophagy [S2]. In the case of neurodegenerative disorders with low levels of autophagy, autophagy activity would need to be increased. Similarly, in infectious diseases autophagy would preferably also be increased to remove pathogens efficiently. In contrast, inhibition of autophagy in established tumors represents a good therapeutic option to overcome chemoresistance and decrease tumor growth [S3], although decreased autophagic activity can promote tumorigenesis [S4]. Therefore, it would be ideal to have agents that either increase or decrease autophagy activity depending on the medical condition of patients. In this context, STK38 inhibitors would require stringent evaluation as STK38 has other cellular functions [S1] besides the role in autophagy described here. In particular, future studies will need to address whether inhibition of STK38 could result in blood borne diseases in humans, since STK38 knock-out mice are prone to develop T cell lymphoma and myeloproliferative disease [S5]. Intriguingly, defective autophagy in hematopoietic stem cells leads to similar immune system diseases [S6], indicating that STK38 null mice might develop lymphoma and/or myeloproliferative disease due to defective autophagy. Consequently, it would be worth re-examining these animals in this respect.

Supplemental Experimental Procedures

Cell lines and transfections

HeLa, HeLa GFP-LC3B, HEK293T, RPE1, and RPE1 GFP-LC3B cells were cultured in RPMI with Glutamax (Gibco), DMEM (Gibco) with 15mM of Hepes (Sigma), or DMEM-F12 with Glutamax (Gibco), respectively. HEK-HT cells were cultured in DMEM medium with 1% L-glutamine (Gibco) and hygromycin 100µg/mL and neomycin 400µg/mL (Invivogen). All media were supplemented with 10% fetal bovine serum (FBS, Gibco) unless otherwise indicated. Cells were cultured at 37°C and 5% CO_2 in a humidified chamber except for U2OS that were cultured at 37°C and 10% CO_2.

Autophagy induction

Cells are plated 2 days (or 3 days for siRNA experiment) before autophagy induction to ensure consistent confluency (below 70%) for experiments. For EBSS-induced autophagy, medium was aspirated, and cells quickly washed once with growth medium (GM) or EBSS (24010-43, Gibco), followed by incubation with GM or EBSS as indicated. For trehalose-induced autophagy, medium was aspirated and immediately replaced with GM containing 100mM trehalose (T0167, Sigma).

***Drosophila* Experiments**

Strains used were: *w1118*, *Cg-Gal4*, *Act5c-Gal4,* *Act5c<FRT>CD2<FRT>Gal4*, *hsflp-1*, *UAS-GFP::Atg8a*, *UAS-mCherry::Atg8a*, *UAS-GFP*, $atg6^{\Delta 1}$ *UAS-mRFP*, *UAS-Trc[S292E]*, *UAS-Trc[S292A+T453A]*, $UAS\text{-}Trc[IR]^{TRiP.JF02961}$. All crosses and experiments were performed at 25°C. Third instar larvae were starved 6h in PBS + 20% sucrose to induce

autophagy. Fat bodies were dissected, mounted in Vectashield (Vectorlabs) and readily imaged on a confocal microscope (Olympus, Leica or Zeiss). For the GFP cleavage assay fat bodies from 10 larvae from each genotype and conditions were dissected and snap frozen before grinding in Laemmli buffer. GFP was detected by immunoblotting with a mouse monoclonal antibody (Roche). For the TUNEL assay wing discs from third instar larvae were dissected in PBS, fixed in 4% PFA in PBS and treated with the kit ApopTag Red (Chemicon) according to the manufacturer instructions. Images were taken using the Zeiss Apotome fluorescent microscope. For the *beclin1/atg6* mutant rescue experiment, third instar larvae were briefly rinsed in PBS and imaged on a Leica MZ16.5 stereoscope equipped with a digital camera.

Yeast Two-Hybrid screens

Yeast two-hybrid (Y2H) screening was performed by Hybrigenics Services (www.hybrigenics-services.com) with full-length human STK38 (Genbank: 31377778) and STK38-PIF [S7] as baits. cDNAs were cloned into pB27 (N-LexA-bait-C fusion) and used to screen a random-primed human fetal brain cDNA library constructed into pP6. More than 70 million clones (7-fold the complexity of the library) were screened with each bait using a mating approach previously described [S8]. Positive colonies were selected on a medium lacking tryptophan, leucine and histidine. Two clones coding for BCN1 were found in the screen with full-length STK38 and one clone in the screen with STK38-PIF.

Reagents, plasmids and siRNAs

Antibodies for Western blots were: LC3B (2775), Beclin1 (3495), cleaved-caspase 3 (9661), RalB (3523), p70(S6K) (2708), phospho-p70(S6K) (Thr389-P) (9206), phospho-ULK1 (Ser757-P) (6888) and Myc (2278) from Cell Signaling. SQSTM1 (PM045), β-Actin (A5441) with Flag (M2) and ULK1 (A7481), Adaptin (610502), GST (Ab6647) and GAPDH (MAB374) were from MBL, Sigma-Aldrich, Becton Dickinson (BD), Abcam and Millipore, respectively. Cleaved-PARP (asp214, 552597), STK38 (H00011329-M011), HA (11867423001) were from BD pharmingen, Abnova and Roche. Anti-phospho-STK38 (Thr444-P) and Mob1 antibodies were previously described [S9, S10]. Anti-HA 12CA5 antibody was used as hybridoma supernatant. For immunofluorescence studies: LC3B (5F10) and ATG12 (CAC-TMD-PH-ATG12) were from Nanotools and Cosmobio. BafilomycinA1, trehalose, tetracycline, 3-MA and EBSS were from Sigma-Aldrich and Gibco. Secondary antibodies donkey anti-mouse-HRP (115-035-003), donkey anti-rabbit-HRP (111-035-045) and donkey anti-rabbit light chain HRP (211-032-171) were from Jackson Immunoresearch and GE Healthcare. Plasmids for mRFP-GFP-LC3B (21074), pcDNA4-Beclin1-HA (24399), pEGFP-ATG14L (21635) and pDs-RED Beclin1 (24405) were from Addgene. STK38 [S9], Exo84-HA [S11], RalB [S12] plasmids were previously described. Vps34-RFP was a kind gift from Ktistakis NT (Babraham Institute, Cambridge, England, UK). siControl non-targeting siRNA (D-001810-01-50) was purchased from Dharmacon. The remaining siRNAs were purchased from Eurogentec or Qiagen:

STK38(1): 5'-CGUCGGCCAUAAACAGCUdTT-3'
STK38(2): 5'-GUAAUAGGCAGAGGAGCAdTT-3'
STK38(3): 5'-CCUUAUCGCUCAACAUGAAdTT-3'

RalA: 5'-GACAGGTTTCTGTAGAAGAdTT-3'
RalB(1): 5'-GAGCCCAGUAUUCACAUUUdTT-3'
RalB(2): 5'-UGACGAGUUUGUAGAAGACdTT-3'
Exo84: 5'-CCACTTTACTCTATATTCAdTT-3'
ULK1(1): 5'-CGCGGUACCUCCAGAGCAAdTT-3'
ULK1(2): 5'-CCCUUUGCGUUAUAUUGUAdTT-3'

The siRNAs targeting Beclin1 [S13] and MOB1A/B [S10] have been described.

Immunofluorescence, time lapse microscopy, and image analysis

For LC3B staining, cells were grown on glass coverslips, washed in PBS, fixed in 4% PFA for 10min, and subjected to cold-methanol treatment for 5min (-20°C). Aldehydes were quenched with 50mM NH_4Cl in PBS for 5min. After PBS washes, fixed cells were incubated in 0.1% Triton X-100 in PBS / 3% BSA for 10min, and then incubated for 30min with anti-LC3B antibody (2μg/mL). Cells were washed, before incubation with anti-mouse Alexa 488 secondary antibody for 30 min, followed by PBS and ddH2O washes and mounting in DAPI-containing mounting medium (Invitrogen). Images were acquired using a confocal microscope (A1R, Nikon). For quantifications, fields were chosen arbitrarily based on DAPI staining, and the number of LC3B dots per cell of at least 50 cells was determined with Image J software. For ATG12 staining, the cold-methanol treatment was skipped and images were acquired using an epifluorescence microscope (Eclipse, 90i, Nikon). Images for ATG14L and PI3P dots quantifications were acquired using an ApoTome 2 and evaluated with ImageJ. Co-localizations studies were carried out on a Leica TCS SP5 confocal microscope. For time lapse videos, images were acquired every 5 min using a spinning disk confocal microscope (Ti-E, Nikon).

Supplemental References

S1. Hergovich, A. (2013). Regulation and functions of mammalian LATS/NDR kinases: looking beyond canonical Hippo signalling. Cell Biosci. *3*, 32.

S2. Choi, J., Jung, W., and Koo, J. S. (2013). Expression of autophagy-related markers beclin-1, light chain 3A, light chain 3B and p62 according to the molecular subtype of breast cancer. Histopathology *62*, 275–286.

S3. White, E. (2012). Deconvoluting the context-dependent role for autophagy in cancer. Nat. Rev. Cancer *12*, 401–410.

S4. Qu, X., Yu, J., Bhagat, G., Furuya, N., Hibshoosh, H., Troxel, A., Rosen, J., Eskelinen, E.-L., Mizushima, N., Ohsumi, Y., et al. (2003). Promotion of tumorigenesis by heterozygous disruption of the beclin 1 autophagy gene. J. Clin. Invest. *112*, 1809–1820.

S5. Cornils, H., Stegert, M. R., Hergovich, A., Hynx, D., Schmitz, D., Dirnhofer, S., and Hemmings, B. A. (2010). Ablation of the kinase NDR1 predisposes mice to the development of T cell lymphoma. Sci. Signal. *3*, ra47.

S6. Mortensen, M., Watson, A. S., and Simon, A. K. (2011). Lack of autophagy in the hematopoietic system leads to loss of hematopoietic stem cell function and dysregulated myeloid proliferation. Autophagy *7*, 1069–1070.

S7. Cook, D., Hoa, L. Y., Gomez, V., Gomez, M., and Hergovich, A. (2014). Constitutively active NDR1-PIF kinase functions independent of MST1 and hMOB1 signalling. Cell. Signal. *26*, 1657–1667.

S8. Fromont-Racine, M., Rain, J. C., and Legrain, P. (1997). Toward a functional analysis of the yeast genome through exhaustive two-hybrid screens. Nat. Genet. *16*, 277–282.
S9. Hergovich, A., Bichsel, S. J., and Hemmings, B. A. (2005). Human NDR kinases are rapidly activated by MOB proteins through recruitment to the plasma membrane and phosphorylation. Mol. Cell. Biol. *25*, 8259–8272.
S10. Hergovich, A., Kohler, R. S., Schmitz, D., Vichalkovski, A., Cornils, H., and Hemmings, B. A. (2009). The MST1 and hMOB1 tumor suppressors control human centrosome duplication by regulating NDR kinase phosphorylation. Curr. Biol. CB *19*, 1692–1702.
S11. Parrini, M. C., Sadou-Dubourgnoux, A., Aoki, K., Kunida, K., Biondini, M., Hatzoglou, A., Poullet, P., Formstecher, E., Yeaman, C., Matsuda, M., et al. (2011). SH3BP1, an exocyst-associated RhoGAP, inactivates Rac1 at the front to drive cell motility. Mol. Cell *42*, 650–661.
S12. Cascone, I., Selimoglu, R., Ozdemir, C., Del Nery, E., Yeaman, C., White, M., and Camonis, J. (2008). Distinct roles of RalA and RalB in the progression of cytokinesis are supported by distinct RalGEFs. EMBO J. *27*, 2375–2387.
S13. Lefort, S., Joffre, C., Kieffer, Y., Givel, A.-M., Bourachot, B., Zago, G., Bieche, I., Dubois, T., Meseure, D., Vincent-Salomon, A., et al. (2014). Inhibition of autophagy as a new means of improving chemotherapy efficiency in high-LC3B triple-negative breast cancers. Autophagy *10*, 2122–2142.
