# Supplementary figures and images for: The Pro-apoptotic STK38 Kinase Is a New Beclin1 Partner Positively Regulating Autophagy
Source: Curr Biol. 2015 Oct 5;25(19):2479–92. doi: 10.1016/j.cub.2015.08.031 (PMC4598746; doi:10.1016/j.cub.2015.08.031)

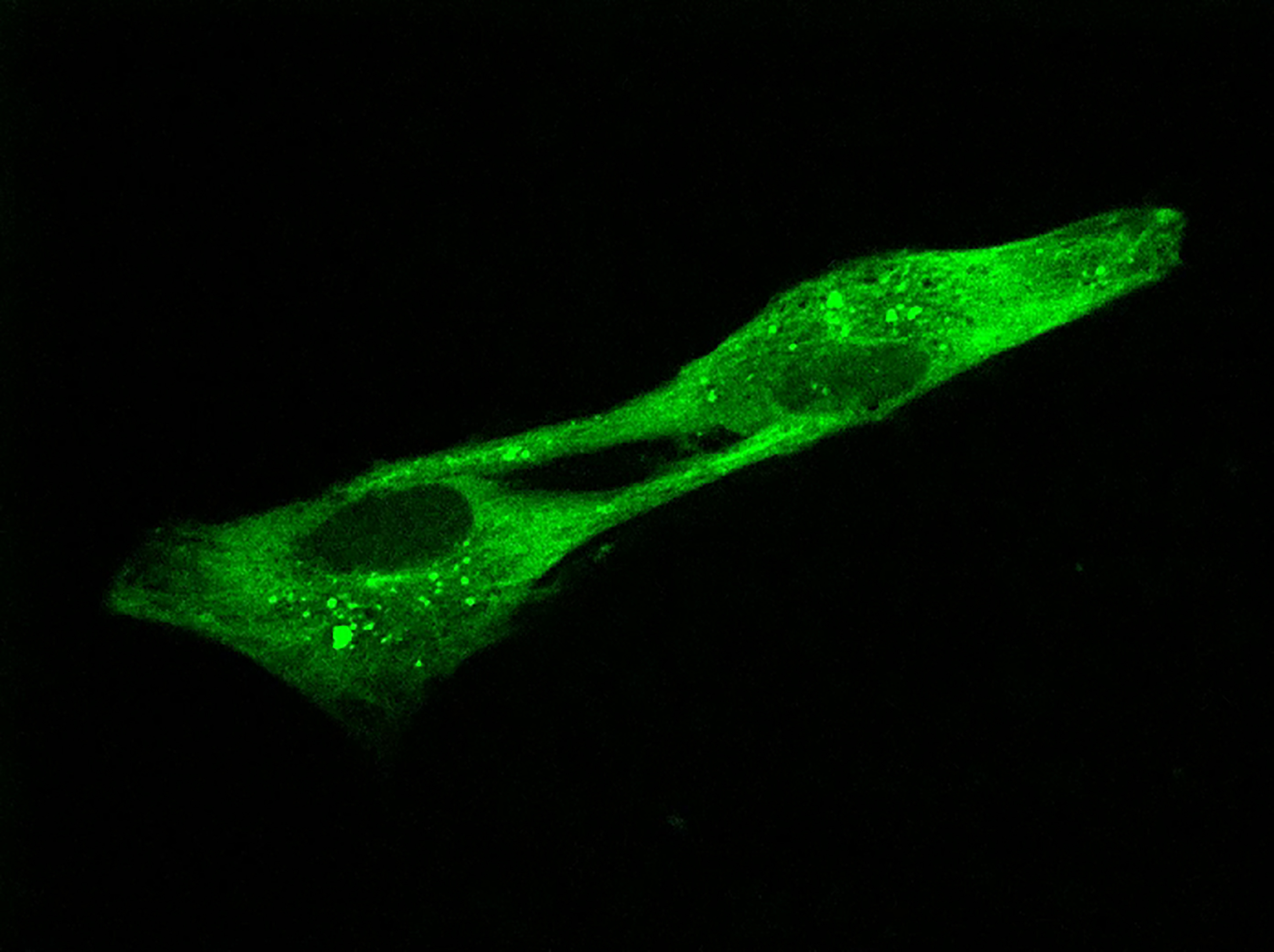

Supplement: Movie S1. Live-Cell Imaging of RPE1-GFP-LC3B Cells upon EBSS Treatment in Control Conditions — Control cells (siControl transfected) were observed for 2 hours. [file mmc2.jpg]

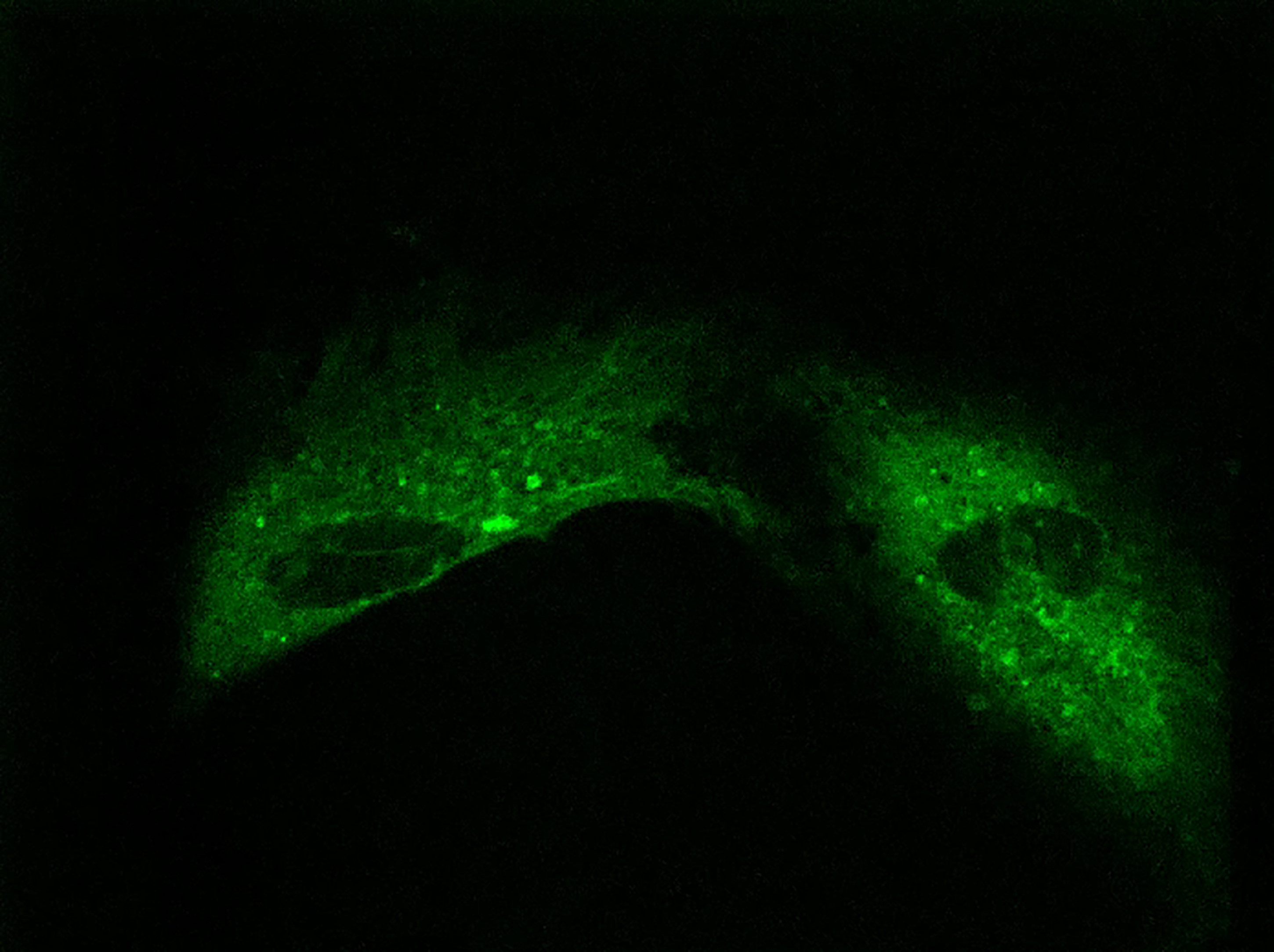

Supplement: Movie S2. Live-Cell Imaging of RPE1-GFP-LC3B Cells upon EBSS Treatment in STK38-Depleted Conditions — STK38-depleted cells (siSTK38 transfected) were observed for 2 hours. [file mmc3.jpg]
