# Supplementary material for: The Pro-apoptotic STK38 Kinase Is a New Beclin1 Partner Positively Regulating Autophagy
Source: Curr Biol. 2015 Oct 5;25(19):2479–92. doi: 10.1016/j.cub.2015.08.031 (PMC4598746; doi:10.1016/j.cub.2015.08.031)
Supplement: Document S2. Article plus Supplemental Information [file mmc4.pdf]

Article

Current Biology

The Pro-apoptotic STK38 Kinase Is a New Beclin1 Partner Positively Regulating Autophagy

Highlights

- STK38 is a novel binding partner of Beclin1, a key regulator of autophagy
- STK38 positively promotes autophagosome formation in human cells and fly larvae
- The activation of the STK38 kinase is regulated during the induction of autophagy
- STK38 and RalB support the coordination between autophagic and apoptotic events

Authors

Carine Joffre, Nicolas Dupont, Lily Hoa, ..., Manolis Fanto, Alexander Hergovich, Jacques Camonis

Correspondence

patrice.codogno@inserm.fr (P.C.),
manolis.fanto@kcl.ac.uk (M.F.),
a.hergovich@ucl.ac.uk (A.H.),
jacquescamonis@gmail.com (J.C.)

In Brief

Autophagy plays key roles in many diseases; hence, it is important to define autophagy regulators whose activities can potentially be modulated. Here, Joffre et al. report the STK38 kinase as a conserved positive regulator of autophagosome formation, assisting cellular fate determination by coordinating autophagic and apoptotic events.

CrossMark

Joffre et al., 2015, Current Biology *25*, 2479–2492
October 5, 2015 ©2015 The Authors
http://dx.doi.org/10.1016/j.cub.2015.08.031

CellPress

Current Biology
Article

CellPress

The Pro-apoptotic STK38 Kinase Is a New Beclin1 Partner Positively Regulating Autophagy

Carine Joffre,[1,5] Nicolas Dupont,[2] Lily Hoa,[3] Valenti Gomez,[3] Raul Pardo,[4] Catarina Gonçalves-Pimentel,[4] Pauline Achard,[5] Audrey Bettoun,[1] Brigitte Meunier,[1] Chantal Bauvy,[2] Ilaria Cascone,[1] Patrice Codogno,[2,*] Manolis Fanto,[4,*] Alexander Hergovich,[3,*] and Jacques Camonis[1,*]
[1]INSERM U830, Institut Curie, Paris 75248, France
[2]INSERM U1151-CNRS UMR 8253, Institut Necker Enfants-Malades, Paris 75993, France
[3]University College London, Cancer Institute, London WC1E 6BT, UK
[4]Department of Basic and Clinical Neuroscience, Kings College London, London SE5 9NU, UK
[5]Cancer Research Center of Toulouse, UMR1037, Toulouse 31037, France
*Correspondence: patrice.codogno@inserm.fr (P.C.), manolis.fanto@kcl.ac.uk (M.F.), a.hergovich@ucl.ac.uk (A.H.), jacquescamonis@gmail.com (J.C.)
http://dx.doi.org/10.1016/j.cub.2015.08.031
This is an open access article under the CC BY license (http://creativecommons.org/licenses/by/4.0/).

SUMMARY

Autophagy plays key roles in development, oncogenesis, cardiovascular, metabolic, and neurodegenerative diseases. Hence, understanding how autophagy is regulated can reveal opportunities to modify autophagy in a disease-relevant manner. Ideally, one would want to functionally define autophagy regulators whose enzymatic activity can potentially be modulated. Here, we describe the STK38 protein kinase (also termed NDR1) as a conserved regulator of autophagy. Using STK38 as bait in yeast-two-hybrid screens, we discovered STK38 as a novel binding partner of Beclin1, a key regulator of autophagy. By combining molecular, cell biological, and genetic approaches, we show that STK38 promotes autophagosome formation in human cells and in *Drosophila*. Upon autophagy induction, STK38-depleted cells display impaired LC3B-II conversion; reduced ATG14L, ATG12, and WIPI-1 puncta formation; and significantly decreased Vps34 activity, as judged by PI3P formation. Furthermore, we observed that STK38 supports the interaction of the exocyst component Exo84 with Beclin1 and RalB, which is required to initiate autophagosome formation. Upon studying the activation of STK38 during autophagy induction, we found that STK38 is stimulated in a MOB1- and exocyst-dependent manner. In contrast, RalB depletion triggers hyperactivation of STK38, resulting in STK38-dependent apoptosis under prolonged autophagy conditions. Together, our data establish STK38 as a conserved regulator of autophagy in human cells and flies. We also provide evidence demonstrating that STK38 and RalB assist the coordination between autophagic and apoptotic events upon autophagy induction, hence further proposing a role for STK38 in determining cellular fate in response to autophagic conditions.

INTRODUCTION

Autophagy is a catabolic process in which cytoplasm bulk, proteins, and organelles are sequestered in autophagosomal vesicles followed by lysosomal degradation [1]. This ensures cellular homeostasis by turning over stable and defective proteins. However, autophagy is not only a sink, as degraded material is recycled [2]. The metabolic state of a cell influences autophagic processes, allowing cells to adapt to poor growth conditions and environmental stresses [3]. Therefore, autophagy studies represent a research area with increasing interest in pathological conditions such as oncogenesis and cancer therapy resistance, as well as cardiovascular, metabolic, and neurodegenerative disorders [4]. The successful manipulation of the autophagic process to improve the management of these pathophysiological disorders requires the identification and characterization of potential drug targets among regulators of autophagy. Recent screens have uncovered new key players in autophagy [5–7]. Many of these key players promote or prevent the initiation of autophagosome formation controlled by the ULK complex (composed of ULK1 or ULK2, FIP200, ATG13, and ATG101) and the Beclin1-PI(3)KC3 (class III phosphatidylinositol 3-kinase) complex (composed of Beclin1, Vps34 and its adaptor Vps15, ATG14L, UVRAG, AMBRA1, and others) [8, 9]. For example, autophagy induction triggers activation of the RalB GTPase and binding to Exo84, a subunit of the exocyst complex. This in turn leads to the recruitment of the Beclin1/Vps34 complex to nascent autophagosomes, thereby promoting autophagosome formation [10].

STK38 (serine-threonine kinase 38), also known as NDR1, belongs to the AGC kinase family being regulated by phosphorylation [11]. Ser281 auto-phosphorylation on the T-loop of STK38 is stimulated by binding of MOB1A/B to an N-terminal regulatory (NTR) domain of STK38, whereas Thr444 phosphorylation in the hydrophobic motif of STK38 is performed by members of the MST kinase family [12]. Phosphorylation of both sites is required for STK38 activation and plays a role in apoptosis and cell-cycle-related processes [12]. STK38 kinases are highly conserved between flies and humans [11], sharing very similar regulatory mechanisms [11, 13, 14]. Noteworthy, human STK38 can even rescue the loss of function of Tricornered (Trc) [14], the fly

CrossMark

counterpart of human STK38, suggesting that human and fly STK38 can share identical cellular functions.

Here, we define STK38 as a novel positive regulator of autophagy. By studying several autophagic markers and events, we show that STK38 depletion severely impairs early autophagosome formation. Our study further revealed that Trc (the fly NDR kinase) is required for autophagy in *Drosophila*, indicating that the autophagic role of STK38 is conserved from flies to humans. Moreover, because hyperactivation of STK38 results in STK38-dependent apoptosis under prolonged autophagy conditions, we discovered that STK38 assists the coordination between autophagic and apoptotic events upon autophagy induction.

RESULTS

STK38 Is a New Binding Partner of Beclin1, a Key Regulator of Autophagy

To identify new binding partners of STK38, two independent yeast-two-hybrid (Y2H) screens were conducted with human full-length STK38 wild-type (WT) or constitutively active STK38-PIF [15] as baits. In both screens, Beclin1, a key regulator of autophagy [8], was identified as binary binding partner of STK38 (Figure S1). Thus, we tested STK38/Beclin1 interactions by co-immunoprecipitation experiments in HEK293 cells, revealing that Beclin1 interacted with STK38 (Figure 1A). Kinase-dead versions of STK38 (K118R or D230N) associated with Beclin1 comparable to STK38(WT) (Figure 1B), indicating that STK38 kinase activity is dispensable for STK38/Beclin1 complex formation. Next, STK38 lacking either the NTR (83–465) or C-terminal (1–380) domains was subjected to co-immunoprecipitations with Beclin1, revealing that Beclin1 binding to N-terminally truncated STK38 was reduced (Figure 1C). Moreover, STK38 versions carrying Y31A or R41A mutations abolishing hMOB1/STK38 complex formation [16] interacted with Beclin1 as efficiently as STK38(WT) (Figure 1D). Thus, the MOB1/STK38 interaction is not a prerequisite for STK38/Beclin1 complex formation. To rule out overexpression artifacts, we examined the interaction between endogenous STK38 and Beclin1 (Figure 1E), showing that Beclin1 co-immunoprecipitates STK38 from HEK293 whole-cell extracts. Additionally, confocal microscopy showed that STK38 and Beclin1 can co-localize in human cells upon autophagy induction (Figure 1F). In EBSS-treated cells, a fraction of STK38 also co-localizes with Vps34 and ATG14L (Figures 1G and 1H), two Beclin1 complex components [8]. A fraction of STK38 further partially co-localized with ULK1 (Figure 1I), a central component of the ULK complex [8, 9]. Taken together, triggered by two independent Y2H screens, we dissected the interaction of STK38 and Beclin1, establishing STK38 as a novel binding partner of Beclin1, a known regulator of early autophagosome formation [8].

STK38 Is a Novel Positive Regulator of Autophagy

Based on the STK38/Beclin1 interaction (Figure 1), we hypothesized that STK38 might be implicated in autophagy. Thus, we analyzed trehalose- and starvation-induced autophagy [17, 18] in STK38-depleted cells (Figure 2). The conversion of LC3B-I into LC3B-II, which correlates with autophagosome numbers [19], was evaluated by immunoblotting, revealing that LC3B-II accumulation was significantly decreased in STK38-depleted cells (Figures 2A, 2B, S2A, and S2B). In parallel, the autophagic flux was analyzed using bafilomycin A1 (BafA1), an inhibitor of autophagic degradation of LC3B-II by blocking autophagosome-lysosome fusion [19]. Consistent with autophagic flux being inhibited in STK38-depleted cells, BafA1 had little effect on LC3B levels (Figures 2A, 2B, S2A, and S2B). To rule out RNAi off-target effects, a rescue experiment was performed using RNAi-resistant STK38 (Figure S2C). Expression of RNAi-resistant STK38(WT) in STK38-depleted cells promoted normal trehalose-induced LC3B-II conversion (Figure S2C), demonstrating that STK38 is required for autophagy. Significantly, STK38 knockdown mirrored defective autophagy induction as observed upon Beclin1 depletion (Figure S2D). Collectively, these findings indicate that STK38 is required for productive autophagy.

To probe the autophagic role of STK38 with an alternative technique, HeLa GFP-LC3B cells [19] were treated with trehalose or EBSS to induce autophagy and the number of autophagosomes marked by GFP puncta was determined (Figure 2C), revealing that autophagosome numbers per cell were significantly decreased upon STK38 knockdown (Figure 2C). Immunofluorescence studies of endogenous LC3B confirmed this observation (Figure 2D). STK38 depletion also impaired trehalose-induced autophagy in untransformed human HEK-HT and RPE1 cells (Figures 2E and 2F), demonstrating that STK38 plays a positive role in autophagy in different human cell lines. Furthermore, we examined levels of p62/SQSTM1, a known autophagy substrate accumulating upon autophagy impairment [20]. Upon EBSS starvation of STK38-depleted cells, p62 levels remained higher than in controls (Figures 2G and S2E), suggesting an impairment of autophagy. In summary, these independent molecular and cellular approaches consistently support a positive role of human STK38 in autophagy.

The Role of STK38/Tricornered in Autophagy Is Conserved in *Drosophila melanogaster*

Because autophagy is conserved among eukaryotes, we next studied autophagy in flies with mutant forms of Trc (CG8637). Trc is the functional fly ortholog of human STK38 [21] because human STK38(WT) can compensate for loss of Trc function [14]. After starvation, which induces autophagy in fly larvae [22], most fat bodies of control larvae (w1118) have lost their regular intracellular structure, and large Atg8/LC3-positive structures together with smaller puncta were observed (Figure 3A, left panels). In contrast, in larvae with Cg-Gal4-driven expression of a Trc RNAi transgene (Trc^{IR}), which can efficiently knockdown Trc levels (Figure S3A), fat bodies mostly preserved their regular appearance with the nuclei in the middle surrounded by dark lipid droplets (Figure 3A, right panels), and the number and size of Atg8-positive structures was significantly lower than in controls (Figures 3B and 3C). As shown in Figures 3A–3C, these results were confirmed by a second experimental approach using larvae expressing a dominant-negative form of Trc ($Trc^{S292A,T453A}$) [14]. In addition, larvae expressing a constitutively active form of Trc (Trc^{S292E}) [14] displayed the opposite phenotype, with increased numbers of large Atg8 puncta when compared to controls (Figures 3A–3C). Significantly, these Atg8 structures were already observed in fed larvae of Trc^{S292E} flies (Figure 3A) and were increased in term of numbers and

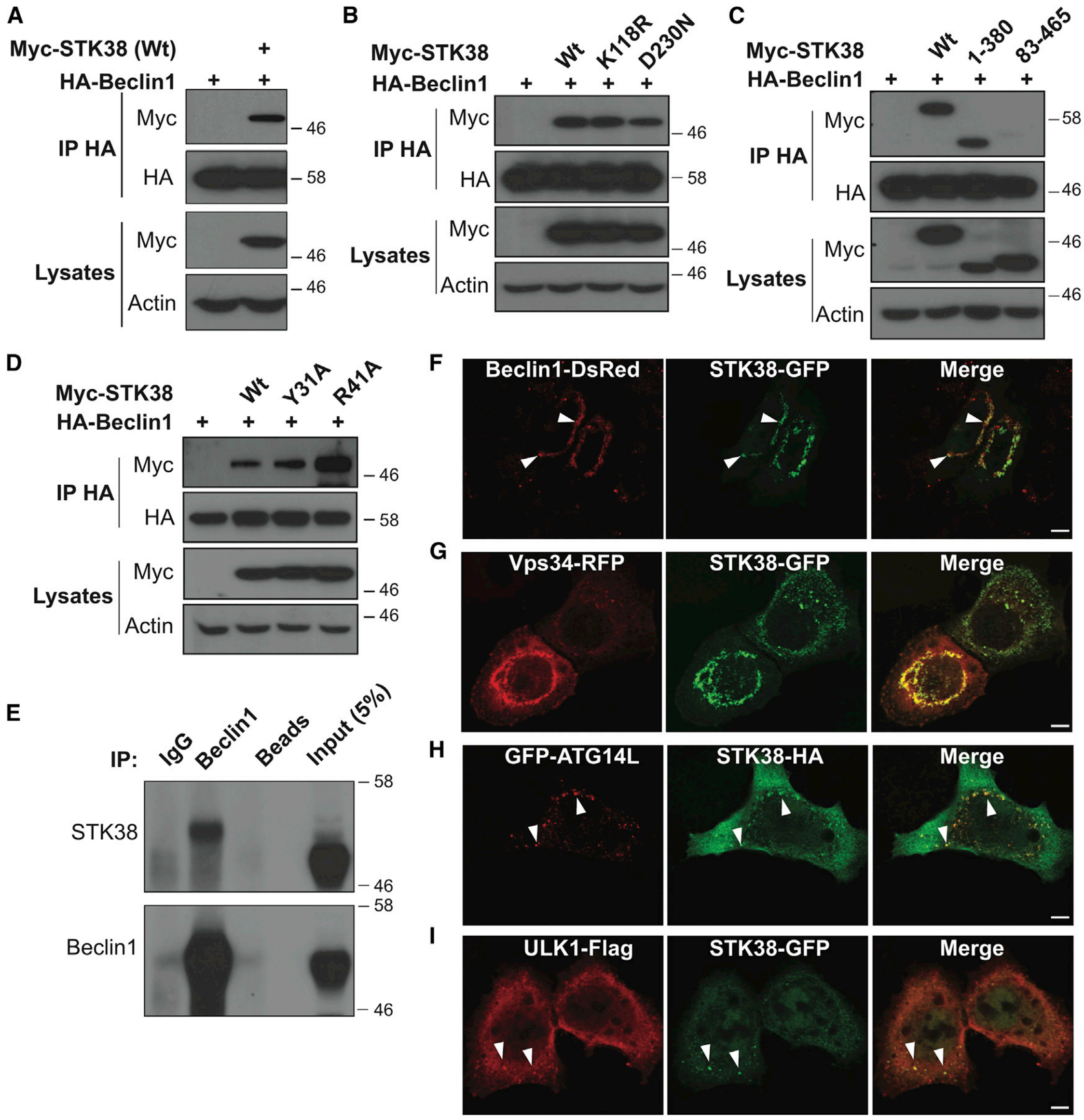

Figure 1. STK38 Is a Novel Binding Partner of Beclin1, a Key Regulator of Autophagy

(A–D) HEK293T cells were co-transfected with indicated vectors expressing Beclin1-HA and selected variants of myc-STK38. D230N, Mg-binding mutant; K118R, ATP-binding mutant; WT, wild-type; Y31A and R41A, MOB1A/B-binding mutants. Beclin1-HA was immunoprecipitated (IP) with anti-HA, and Beclin1-associated STK38 was detected with anti-myc. Input lysates are shown.

(E) Endogenous Beclin1 was IP with anti-Beclin1 antibody, and Beclin1-associated STK38 was detected by western blotting. Beads, control sepharose beads only; IgG, control antibody.

(F–I) HeLa cells were co-transfected with indicated plasmids. Twenty-four hours later, cells were subjected to EBSS treatment for 2 hr, fixed, and stained. Representative confocal pictures are shown. White arrowheads indicate co-localizations between STK38 and Beclin1-ATG14L-Vps34 complex components or ULK1. The scale bars represent 5 μM.

size (Figures 3B and 3C), suggesting that elevated Trc activity is sufficient to increase autophagosome formation in this setting. To exclude the possibility that the observed effects are due to different genetic backgrounds, we performed mosaic studies with the Trc^{IR} line. In starved larvae, cells expressing the Trc RNAi transgene (marked by GFP) did not form autophagosomes, whereas their neighboring cells displayed large Atg8 punctae (Figure 3D), further supporting our notion that Trc is critical for autophagosome formation in fly larvae.

To validate the autophagic role of Trc with a different method, we performed a GFP cleavage and accumulation assay by studying the GFP::Atg8a transgene in the presence or absence of Trc manipulations. As expected, in fat bodies collected from WT larvae, GFP readily accumulated upon starvation (Figure 3E), indicating increased autophagy activity upon starvation. Contrarily, in samples collected from the Trc^{IR} line, the GFP levels barely increased, accompanied by lower levels in fed conditions (Figure 3E). In larvae expressing activated Trc^{S292E}, the increase

CellPress

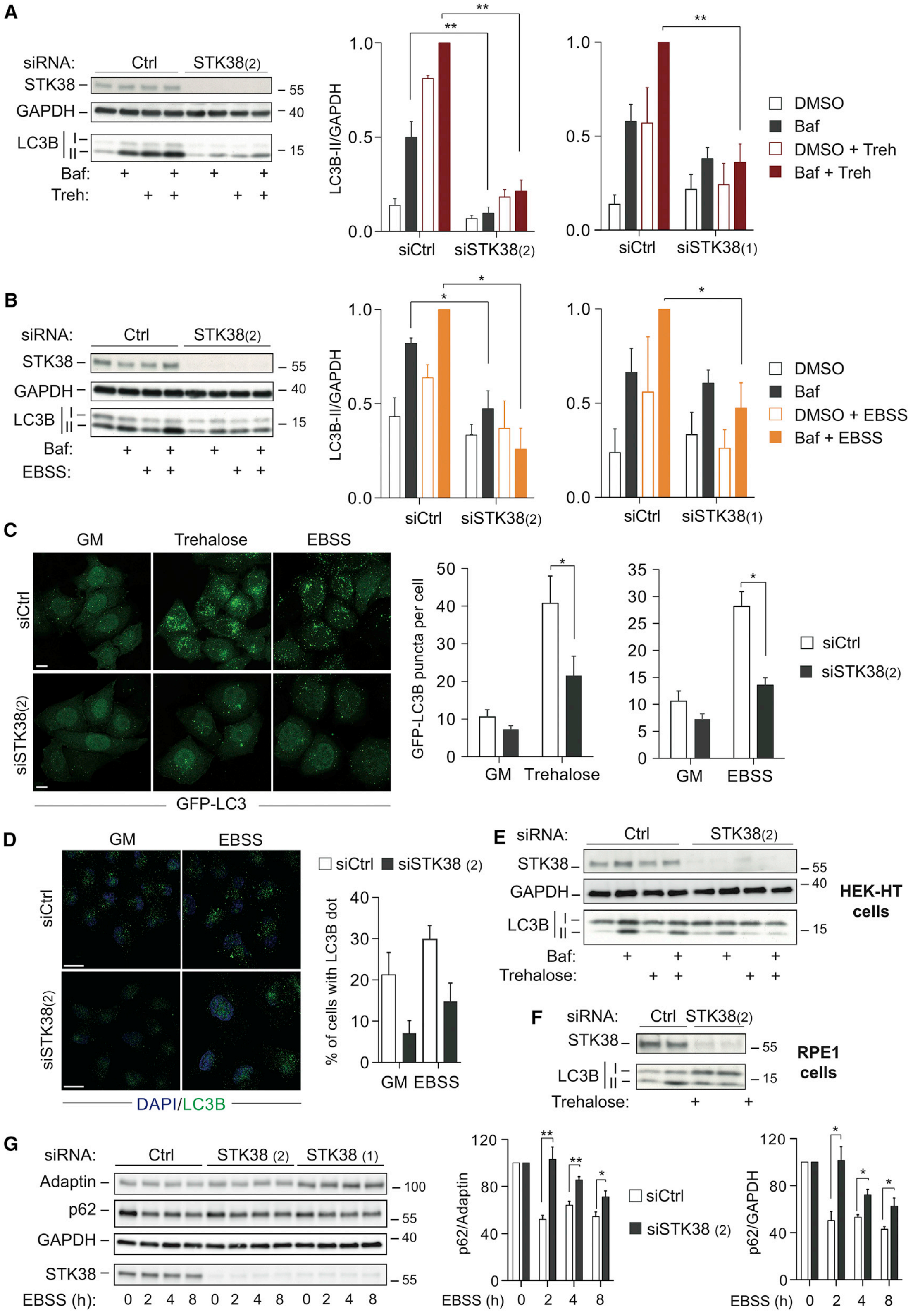

Figure 2. STK38 Is a New Positive Regulator of Autophagy

(A and B) HeLa cells were transfected with indicated siRNAs. Seventy-two hours later, cells were incubated with trehalose for 16 hr (A) or EBSS for 4 hr (B) with or without bafilomycinA1 (Baf) for 4 hr, followed by immunoblotting using indicated antibodies (left panels). Histograms represent LC3B-II/GAPDH ratios obtained by densitometric analysis (n = 3 ± SEM) of western blots (right panels).

(legend continued on next page)

in GFP was more pronounced upon starvation and the levels in fed conditions were also elevated compared to WT samples (Figure 3E). Collectively, these data described in Figure 3 support our conclusion that Trc is necessary for starvation-induced autophagy in flies.

Finally, we used fly genetics to determine whether Trc can function upstream or downstream of Atg6 (fly Beclin1). Mutant larvae for *atg6* display melanotic blood cell mass formation and die at the third instar stage [23]. Thus, we wondered whether expression of activated Trc^{S292E} would be sufficient to compensate for *atg6* loss of function in this context. Significantly, *atg6* mutant larvae expressing activated Trc displayed decreased formation of blood cell mass in contrast to control *atg6* mutant animals (Figures S3B and S3C), suggesting Trc can function downstream of Atg6.

Together with Figure 2, these data demonstrate that STK38 kinases are conserved regulators of autophagy in flies and humans, further proposing that Beclin1 can function upstream of STK38.

STK38 Is Required for Early Autophagic Events

Based on the results presented in Figures 1 and 2, we hypothesized that STK38 is implicated in autophagosome formation rather than later autophagic steps such as autophagosome-lysosome fusion. To probe this hypothesis, we performed time-lapse experiments using RPE1-GFP-LC3B cells (Figures 4A and 4B; Movies S1 and S2). In basal autophagic conditions, autophagosome numbers decreased upon STK38 knockdown (Figures 4A and 4B). Upon EBSS treatment, the formation of intense GFP dots gradually increased over time in controls, whereas in STK38-depleted cells, autophagosome numbers did not change significantly (Figures 4A and 4B), illustrating that STK38 depletion severely impaired autophagosome formation. Alternatively, we evaluated LC3B-II accumulation upon EBSS starvation in the presence of BafA1 (Figures 4C and 4D). In controls, as expected, LC3B-II progressively accumulated upon prolonged starvation when combined with BafA1. In contrast, LC3B-II accumulation was decreased in STK38-depleted cells (Figures 4C and 4D). Taken together, these experiments (Figures 4A–4D) strongly suggest a role for STK38 in early steps of autophagosome formation.

To further expand on the role of STK38 in autophagosome formation, we monitored the subcellular localization of ATG14L, WIPI-1, and ATG12 (Figures 4E, 4F, and S4). ATG14L is needed for autophagosome biogenesis [8]. WIPI-1 and ATG12 are present on pre-autophagosomes [1]. Therefore, these approaches allowed us to study newly formed autophagosomes. First, we confirmed that STK38 was also required for autophagosome formation in U2OS cells upon EBSS treatment (Figure S4A), as observed in HeLa, HEK-HT, and RPE1 cells (Figure 2). Then, we assessed the number of GFP-WIPI-1 puncta in U2OS GFP-WIPI-1 cells, revealing that the number of WIPI-1 puncta was considerably reduced upon STK38 knockdown (Figures S4B and S4C). In EBSS-starved HeLa, the percentage of cells displaying GFP-ATG14L dots was also significantly reduced in STK38-depleted cells (Figures 4E and 4F). Similar results were obtained when endogenous ATG12 was examined (Figures S4D and S4E). Based on the evaluation of PI3P dots in EBSS-starved cells as described [24], we further concluded that Vps34 activity was decreased upon STK38 depletion (Figures 4G and 4H). Collectively, these data along with the observation that STK38 associates with Beclin1 (Figure 1), a key regulator of vesicle nucleation [8], are consistent with STK38 regulating either the induction or vesicle nucleation stages during early autophagosome formation.

To test whether STK38 may also have a role in subsequent autophagy events like the fusion between autophagosomes and lysosomes, we used the mRFP-GFP-LC3B tandem probe [25]. This dual-color analysis enables a direct assessment of the level of autophagosome-lysosome fusion events and permits one to distinguish between autophagosomes (yellow) and autophagolysosomes (red) [19]. This approach revealed that, upon starvation, in spite of a total reduction of autophagosomes by 50% in STK38-depleted cells, the ratio between yellow and red signals remained unaffected (Figures S4F and S4G). Because a defect in fusion of autophagosomes with lysosomes would manifest by an accumulation of yellow dots (autophagosomes) with decreased red (autophagolysosomes) signals, these data are in agreement with a role for STK38 in early autophagosome formation rather than maturation.

STK38 Supports the Interaction of Beclin1 and RalB with Exo84

One key event promoting early autophagosome formation is the RalB-mediated formation of Beclin1/Exo84 complexes [10]. Given our findings that STK38 is a novel binding partner of Beclin1 (Figure 1) and regulator of early autophagic events (Figures 2, 3, and 4), we hypothesized that STK38 might play a role in regulating RalB/Exo84 interactions, which are known to facilitate the recruitment of the Beclin1/Vps34 complex to nascent autophagosomes by supporting Beclin1/Exo84 complex formation [10, 26, 27]. Therefore, we assessed these interactions by co-immunoprecipitation experiments in control and

(C) HeLa GFP-LC3B cells were transfected with indicated siRNAs. Seventy-two hours later, cells were treated with trehalose for 16 hr or EBSS for 2 hr and processed for immunofluorescence analysis. Representative confocal pictures (left panels; scale bar, 10 μM) are shown. Histograms (right panels) represent the number of GFP-LC3B puncta per cell (n = 3 ± SEM).

(D) HeLa cells were transfected with indicated siRNAs. Seventy-two hours later, cells were subjected to EBSS treatment for 2 hr, fixed, and stained for LC3B and DAPI. Representative confocal pictures are shown. The scale bar represents 10 μM. Histogram shows the quantification of the percentage of cells displaying LC3B dots performed with the Image J software on at least 50 cells through a minimum of five pictures.

(E and F) HEK-HT and RPE1 cells were transfected with indicated siRNAs. Seventy-two hours later, cells were treated with trehalose with or without bafilomycinA1 (Baf). LC3B-II levels were assessed by western blots.

(G) HeLa cells were transfected with indicated siRNAs. Seventy-two hours later, cells were subjected to EBSS treatment as indicated. p62 level was assessed by western blotting (left panel). Histograms show p62/adaptin and p62/GAPDH ratios obtained by densitometric analysis ($n \geq 3 \pm$ SEM) of western blots (right panel). To determine statistically significant differences, unpaired two-tailed Student's t tests were carried out (*$p < 0.05$; **$p < 0.01$).

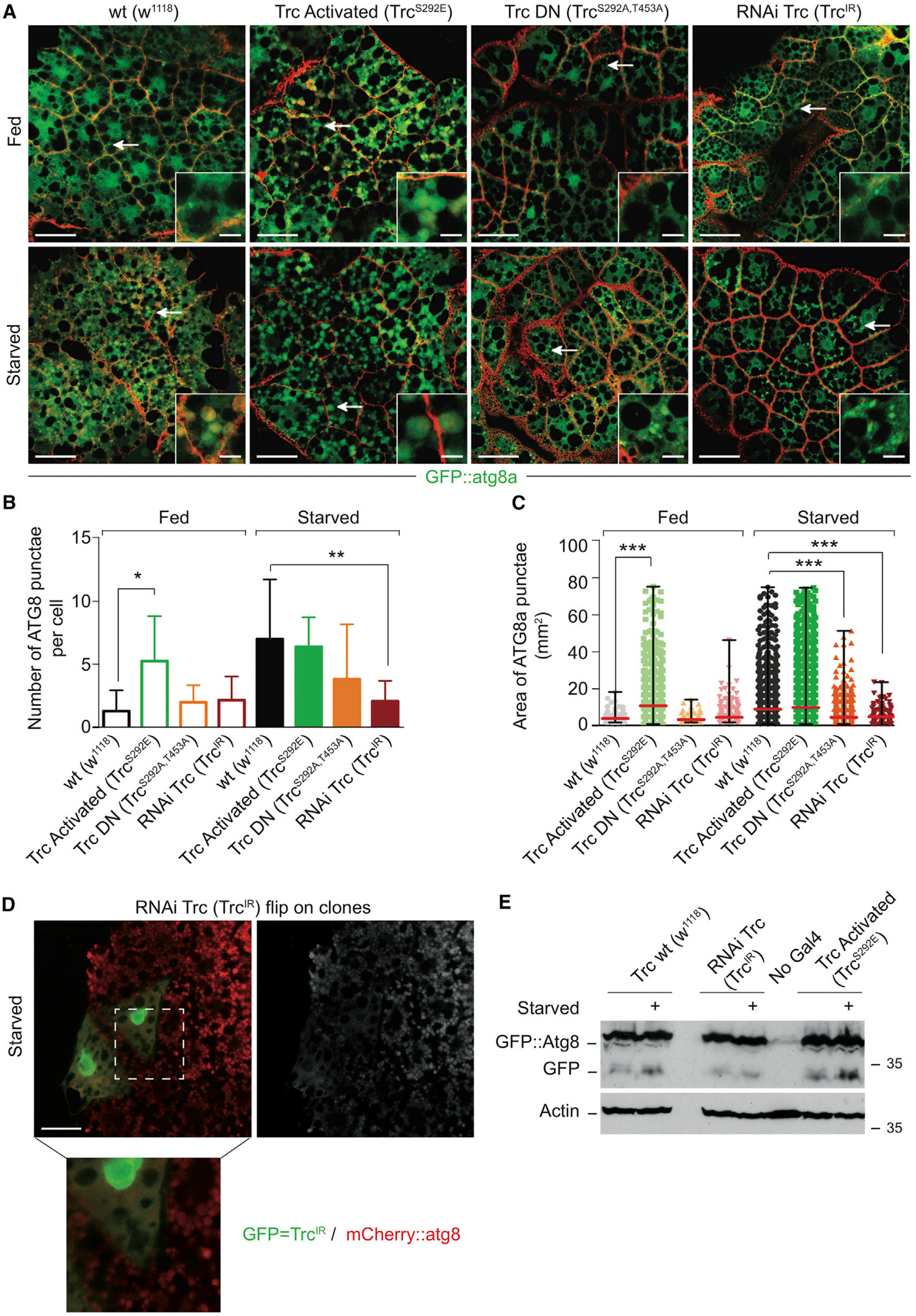

Figure 3. The Fly STK38 Kinase Tricornered Is Required for Autophagy in Larvae of *Drosophila melanogaster*

(A) Single confocal scans of fat bodies from fed or starved larvae of different *trc* transgenic alleles and control strain (w^{1118}). GFP::Atg8a is shown in green, and membrane-bound RFP is red. The scale bar represents 50 μM. Bottom right insets represent magnifications of areas indicated by white arrows. The scale bar in magnifications represents 10 μM.

(legend continued on next page)

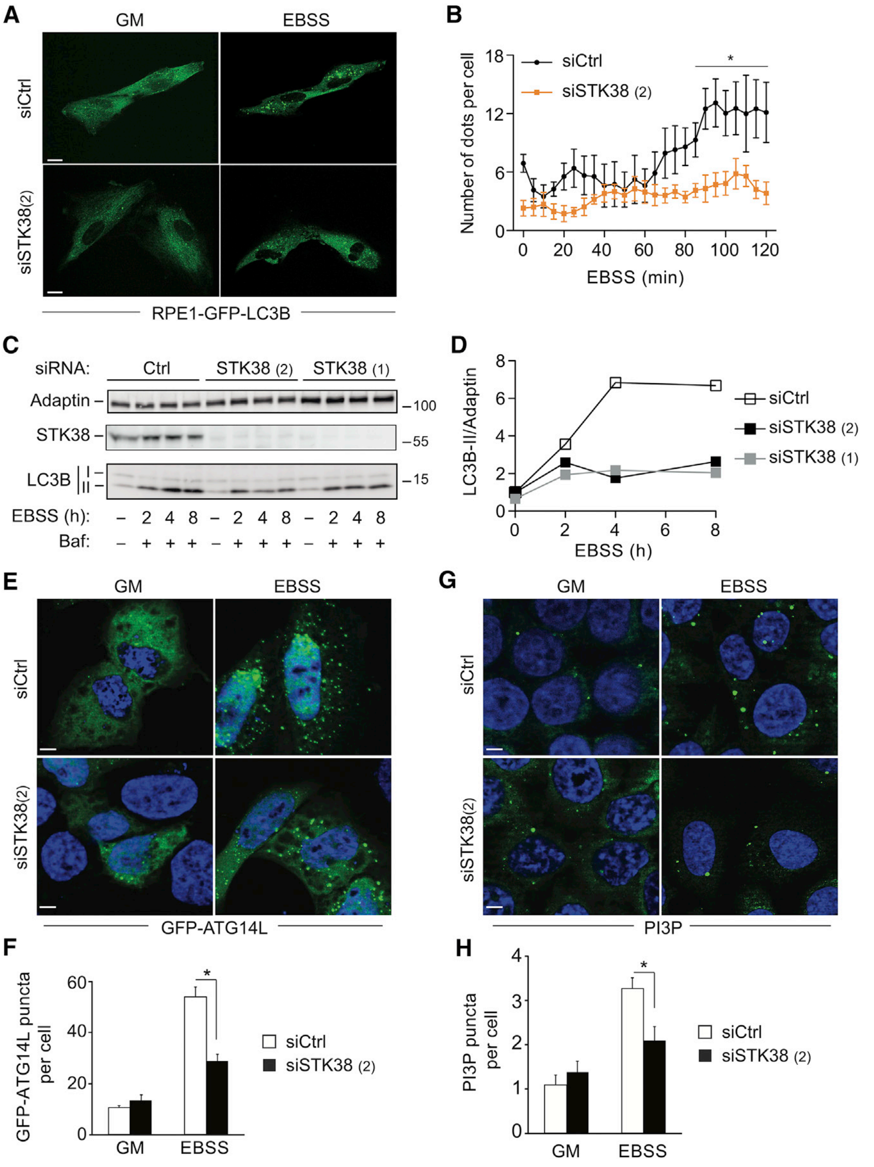

Figure 4. STK38 Plays a Role in Early Autophagosome Formation

(A) RPE1-GFP-LC3B cells were transfected with indicated siRNAs. Seventy-two hours later, cells were treated with EBSS for 2 hr and subjected to time-lapse video microscopy. Representative images (before and end of EBSS treatment) are shown. The scale bar represents 10 μM. See Movies S1 and S2 for the entire video sequence.

(B) Quantifications for experiments in (A) performed on at least 12 cells spread on a minimum of five fields. Graph represents the number of LC3B dots per cell during EBSS treatment.

(C) HeLa cells were transfected with indicated siRNAs. Seventy-two hours later, cells were treated with EBSS as indicated (± bafilomycinA1; Baf), and processed for western blotting.

(D) Quantification of experiment shown in (C). Graphs represent LC3B-II/adaptin ratios.

(E) HeLa cells were transfected with indicated siRNAs. Forty-eight hours later, cells were transfected with GFP-ATG14L and treated the following day with EBSS for 10 min.

(F) Quantifications for experiments shown in (E). Graph represents the number of GFP-ATG14L dots per cell (n = 3 ± SEM).

(G) HeLa cells were transfected with indicated siRNAs. Seventy-two hours later, cells were treated with EBSS for 10 min. To measure PI3P dots formation (Vps34 activity), cells were fixed and PI3P was marked with recombinant FYVE-FYVE^{GST} protein, followed by FITC-conjugated anti-GST antibody.

(H) Quantifications for experiments shown in (G). Graph represents the number of PI3P dots per cell (n = 3 ± SEM). To determine statistically significant differences, unpaired two-tailed Student's t tests were carried out (*p < 0.05).

STK38-depleted cells (Figure 5). As reported [10], EBSS starvation increased the association of Exo84 with RalB in controls (Figure 5A). In contrast, the Exo84/RalB interaction was diminished in starved STK38-depleted cells (Figure 5A). Notably, this interaction was also decreased at basal conditions (Figure 5A). Similarly, the binding of Exo84 to constitutively active RalB G23V was reduced in STK38-depleted cells at basal conditions (Figure 5B), suggesting that STK38 is needed to support the autophagy-driving interaction between Exo84 and RalB. In full support of these observations, our analysis of STK38-depleted cells further revealed that STK38 is also required to promote the Exo84/Beclin1 interaction (Figure 5C). As reported [10], EBSS starvation increased the association of Exo84 with Beclin1 in controls (Figure 5C). However, in STK38-depleted cells, Exo84/Beclin1 complex formation was reduced under basal and starvation conditions (Figure 5C). Collectively, these results suggest that STK38 is necessary for efficient Exo84/Beclin1 and Exo84/RalB complex formations, which are essential for autophagy induction during nutrient restriction. This mechanistic insight further strengthens our model, in which STK38 promotes early autophagosome formation (Figure 5D).

(B and C) Quantification of the number (B) and size (C) of GFP::Atg8a-positive structures. Histograms show data from approximately 150 cells (fed) and 400 cells (starved) analyzed with ImageJ software. For statistical analysis, one-tail unpaired t test (B) and one-way ANOVA followed by post hoc Bonferroni's multiple comparison test (C) were performed (*p < 0.05; **p < 0.01; ***p < 0.001).

(D) Single confocal section of a flip-on clone for the *Trc^IR* transgene in the fat body of a starved larva. Cells expressing the Trc RNAi transgene are labeled by GFP (green). mCherry::atg8 is shown in red. The scale bar represents 30 μM. The bottom panel represents a higher magnification image of the area marked in the image above.

(E) GFP cleavage assay in fat bodies collected from fed or starved larvae expressing indicated transgenic alleles together with *Actin5c-Gal4*. Control strain (w^{1118}) is shown. Levels of GFP::Atg8a and cleaved GFP were detected by western blot. Actin served as loading control.

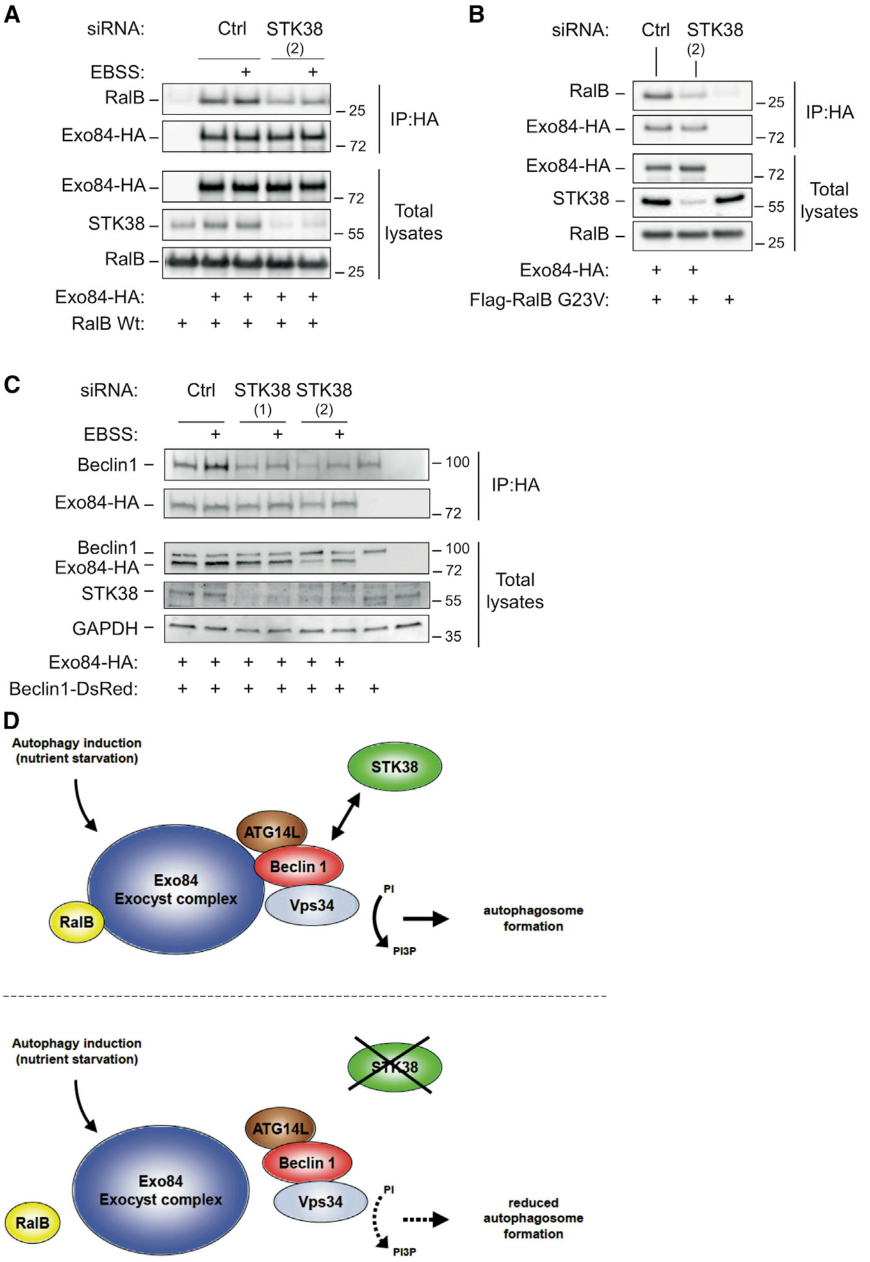

Figure 5. STK38 Supports Exo84/Beclin1 and Exo84/RalB Complex Formation

(A–C) HEK293T cells were transfected with indicated siRNAs. Forty-eight hours later, cells were transfected with indicated vectors and processed the following day for Exo84-HA IP using anti-HA. In (A) and (C), indicated transfected cells were treated with EBSS for 90 min before processing for IP. Exo84-associated RalB or Beclin1 were detected by western blotting. Total input lysates are shown.

(D) Working model of STK38-dependent induction of autophagy. STK38 loss of function interferes with Exo84/RalB and Exo84/Beclin1 interactions, which are required to efficiently initiate PI3P formation by the Beclin1-ATG14L-Vps34 complex. Therefore, upon STK38 depletion, PI3P levels are lower and ATG14L recruitment to autophagosomes is impaired, consequently resulting in reduced autophagosome formation.

STK38 Is Activated upon Induction of Autophagy

Given that our findings established STK38 as a novel autophagy player (Figures 1, 2, 3, 4, and 5), we wondered whether STK38 is regulated upon autophagy induction (Figure 6). To monitor endogenous STK38 activity, we used an antibody raised against phosphorylated Thr444 (Thr444-P), considering that Thr444 phosphorylation is essential for and reflects STK38 activity [12]. In full support of a function of STK38 in autophagy, trehalose and EBSS treatments induced STK38 activation (Figures 6A–6C). To explore this regulation further, we pursued three avenues. First, we compared the effect of WT and kinase-dead STK38 (Figures 6D, 6E, and S5A). Second, we examined the involvement of MOB1 (Figures 6F and 6G), a known STK38 co-activator needed for Thr444 phosphorylation in cells [28]. Third, we studied whether STK38 activation requires early autophagic processes (Figures 6H, 6I, S5, and S6).

Overexpression of kinase-dead (kd) STK38 inhibited trehalose- or EBSS-induced LC3B-II accumulation, whereas cells overexpressing STK38(WT) displayed no statistically significant difference (Figures 6D, 6E, and S5A). Because STK38(kd) can function as a dominant-negative form [29], this finding complements our previous RNAi-mediated loss of function approaches of STK38 (Figures 2, 3, and 4). Moreover, we assessed whether MOB1 is required for Thr444 phosphorylation and autophagy in this setting, revealing that MOB1 depletion reduced autophagy-induced STK38 phosphorylation, paralleled by an impairment of starvation-induced LC3B-II accumulation (Figures 6F and 6G). In summary, these results indicate that STK38 is rapidly activated upon autophagy induction and requires its co-activator MOB1 for supporting autophagy.

Next, given that EBSS and trehalose treatments resulted in a rapid rise of STK38 phosphorylation (Figures 6A–6C), we wondered whether inhibition of early autophagy events would affect STK38 activation. Therefore, we interfered with autophagy induction on four different levels. First, we depleted cells of RalB, which blocks EBSS and trehalose-induced autophagy (Figures S5B and S5C) [10]. Second, Exo84 was depleted, because this exocyst component is crucial for autophagy induction [10]. Third, we blocked early autophagosome formation by interfering with the PI(3)KC3 complex by either treating cells with 3-methyladenine (3-MA), which inhibits Vps34 [24] or depleting Beclin1. Fourth, ULK1 was depleted, because the ULK complex is critical for activation of the Beclin1-PI(3)KC3 complex [30, 31]. Significantly, these approaches revealed that blocking autophagy by

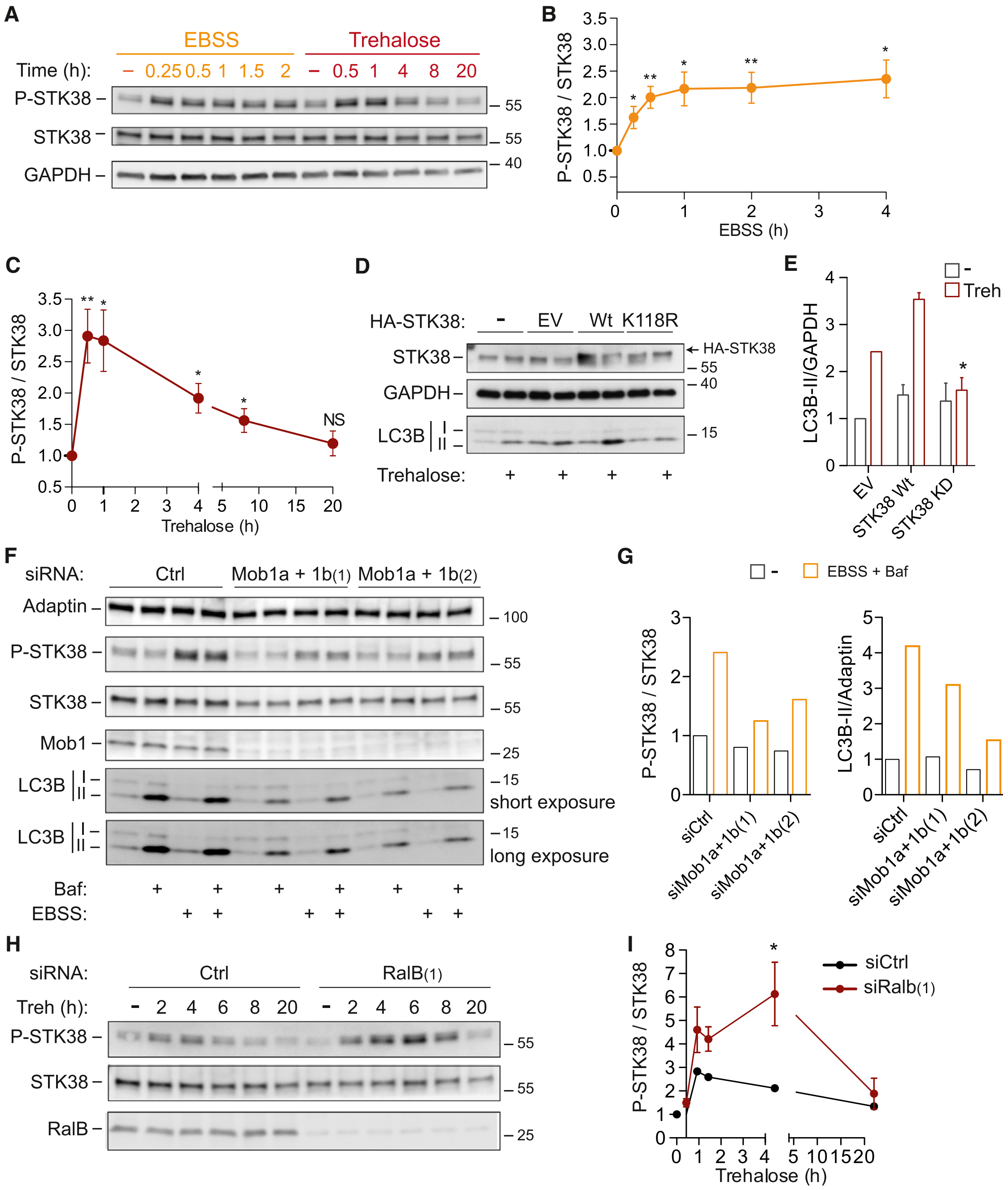

Figure 6. The STK38 Kinase Is Activated upon Autophagy Induction

(A) HeLa cells were treated with EBSS or trehalose as indicated, before processing for western blot analysis.

(B and C) Quantification of experiments shown in (A). Graphs represent phospho-STK38/total STK38 ratios (n = 4 ± SEM).

(D) HeLa cells were transfected with indicated plasmids. Twenty-four hours later, cells were treated with trehalose for 8 hr and processed for western blot analysis. EV, empty vector; K118R, kinase-dead. See also Figure S6.

(E) Quantification of experiments shown in (D). Histogram shows LC3B-II/GAPDH ratios (n = 3 ± SEM).

(F) HeLa cells were transfected with indicated siRNAs. Seventy-two hours later, cells were treated with EBSS for 2 hr ± bafilomycinA1 (Baf) and processed for western blot analysis.

(G) Quantification for experiment shown in (F). Histograms show phospho-STK38/total STK38 ratios (left panel) and LC3B-II/adaptin ratios (right panel).

(H) HeLa cells were transfected with indicated siRNAs (B). Seventy-two hours later, cells were treated with trehalose for the indicated times, followed by processing for immunoblotting using indicated antibodies.

(I) Quantification of experiments shown in (H). The graph shows the phospho-STK38/total STK38 ratios obtained by densitometric analysis of western blots (at least n = 3 ± SEM). To determine statistically significant differences, unpaired two-tailed Student's t tests were carried out (*$p < 0.05$; **$p < 0.01$).

Exo84, Beclin1, ULK1 depletion, or 3-MA treatment impaired STK38 phosphorylation upon EBSS starvation (Figures S5 and S6), whereas in RalB-depleted cells, STK38 activation was elevated when compared to controls (Figures 6H, 6I, and S5). In contrast, upon nutrient starvation, the phosphorylations of p70(S6K) and ULK1 mediated by mTOR [32, 33] decreased in STK38-depleted cells comparable to controls (Figures S6C and S6D). These findings cumulatively suggest that ULK1 and Beclin1 are likely to act upstream of STK38 upon autophagy induction, whereas STK38 appears to be dispensable for the suppression of mTOR activity upon nutrient deprivation.

Intriguingly, upon prolonged autophagy induction, increased STK38 phosphorylation in RalB-depleted cells was paralleled by augmented levels of cleaved PARP (Figure S5), suggesting that the observed increase of STK38 phosphorylation upon RalB depletion could be related to the reported pro-apoptotic role of STK38 [34]. Collectively, these observations indicate that autophagy induction as a consequence of nutrient starvation is required for STK38 activation. Moreover, we were tempted to speculate that RalB depletion combined with autophagy induction might trigger activation of STK38 to drive apoptosis.

STK38 and RalB Support the Balance between Autophagy and Apoptosis

Considering that, upon autophagy induction, STK38 was hyperactivated in RalB-depleted cells (Figures 6 and S5), we hypothesized that RalB-STK38 signaling might contribute to the important interplay between autophagy and apoptosis [35]. To test this hypothesis, we expanded our analysis of RalB-depleted cells in EBSS starvation conditions (Figure 7). Despite blocked autophagy upon RalB depletion (Figures S5B and S5C), STK38 phosphorylation dramatically increased upon prolonged EBSS treatment (Figure 7A). In parallel to elevated STK38 activation, RalB depletion also caused apoptosis as judged by the accumulation of cleaved PARP and caspase 3 (Figure 7A). These findings suggested that elevated STK38 activity might have led to apoptosis of RalB-depleted cells. Indeed, STK38 depletion alone or co-depletion of RalB and STK38 abrogated EBSS-induced PARP cleavage (Figures 7B and 7C), indicating that STK38 activation upon autophagy induction was triggering apoptosis in the absence of RalB. Overexpression of constitutively active STK38-PIF [15] was sufficient to enhance EBSS-induced PARP cleavage without RalB depletion (Figure S7A), suggesting that elevated STK38 activity is sufficient to trigger apoptosis. In full support of this interpretation, we also observed elevated levels of apoptosis in *Drosophila* larval imaginal wing discs upon expression of activated Trc^{S292E} (Figures S7B and S7C). Collectively, these observations support our notion that, upon autophagy induction, the activation of STK38 must be tightly regulated to prevent pre-mature apoptotic signaling.

To monitor apoptosis by an independent method, annexin V/PI stainings were quantified by flow cytometry to visualize apoptotic cells (Figure 7D). In RalB-depleted cells, the number of annexin-V-positive cells was dramatically increased after EBSS treatment, whereas co-depletion of RalB and STK38 reverted this apoptotic phenotype (Figure 7D). Next, to rule out the possibility that STK38 phosphorylation in RalB-depleted cells was due to RalA activation, we silenced RalA alone (Figure 7E) or co-depleted RalA and RalB (Figure 7F). This revealed that RalA depletion causes a slight decrease in STK38 phosphorylation when compared to control cells (Figure 7E), whereas co-depletion of RalA and RalB resulted in the same phenotype as observed upon RalB depletion only (compare Figures 7A and 7F), namely STK38 overactivation and increased PARP cleavage upon prolonged EBSS starvation. This final set of data showed that RalA is not required and not responsible for STK38 hyperactivation upon RalB depletion during autophagy induction.

Collectively, these findings indicate that STK38 activation is enhanced upon RalB knockdown combined with autophagy induction, leading to increased apoptosis in a STK38-dependent manner. Therefore, in the absence of RalB, STK38 activity fails to be directed toward autophagy induction, which instead triggers apoptosis in response to autophagic stimuli. Our data would therefore suggest that RalB normally serves to limit STK38 activation and apoptosis induction upon nutrient deprivation, indicating that STK38 and RalB, in addition to their roles in autophagy, also support the balance between autophagy and apoptosis.

DISCUSSION

Here, we define the pro-apoptotic STK38 kinase as a new Beclin1-binding partner. Therefore, we studied STK38 in autophagy, unveiling STK38 as a novel positive regulator of autophagy. Specifically, STK38 depletion severely impaired autophagosome formation. Upon autophagy induction, STK38 is phosphorylated on Thr444, a key residue for STK38 activation, suggesting that STK38 is activated upon autophagy. Our study further revealed that Trc, the fly counterpart of STK38, is required for normal autophagy in fly larvae, strongly indicating that the positive role of STK38 kinase in autophagy is conserved from flies to humans. Finally, we found that STK38 as pro-apoptotic kinase supports the balance between autophagy and apoptosis upon nutrient deprivation.

Cumulatively, our evidence strongly suggests that STK38 is required for early steps of autophagosome formation. In STK38-depleted cells, LC3B-II conversion assays coupled with time-lapse experiments clearly showed that autophagosome biogenesis is reduced (Figures 2 and 4). By using the mRFP-GFP-LC3B tandem probe, we discriminated between newly formed autophagosomes and autophagolysosomes (Figure S4), showing that STK38 depletion does not negatively impact autophagosome-lysosome fusion. Moreover, ATG14L, ATG12, and WIPI-1 puncta numbers were decreased in STK38-depleted cells. In full agreement with these observations, Vps34 activity was significantly reduced upon STK38 depletion (Figure 4). Furthermore, we observed that STK38 supports Exo84/Beclin1 and Exo84/RalB complex formations (Figure 5), which are important for initiating autophagosome formation [10]. Overexpression of dominant-negative STK38/Trc also inhibited autophagy (Figures 3 and 6), further supporting our RNAi-based results. Therefore, all our evidence collectively proposes a model in which STK38 is required for early steps in autophagosome formation (Figure 5D).

In full agreement with our findings on human STK38 (Figures 2 and 4), Trc, the fly counterpart of STK38, is required for autophagy in flies (Figure 3). Upon starvation to induce autophagosome formation, larvae expressing either Trc RNAi or dominant-negative Trc (Trc^{S292A,T453A}) displayed an altered

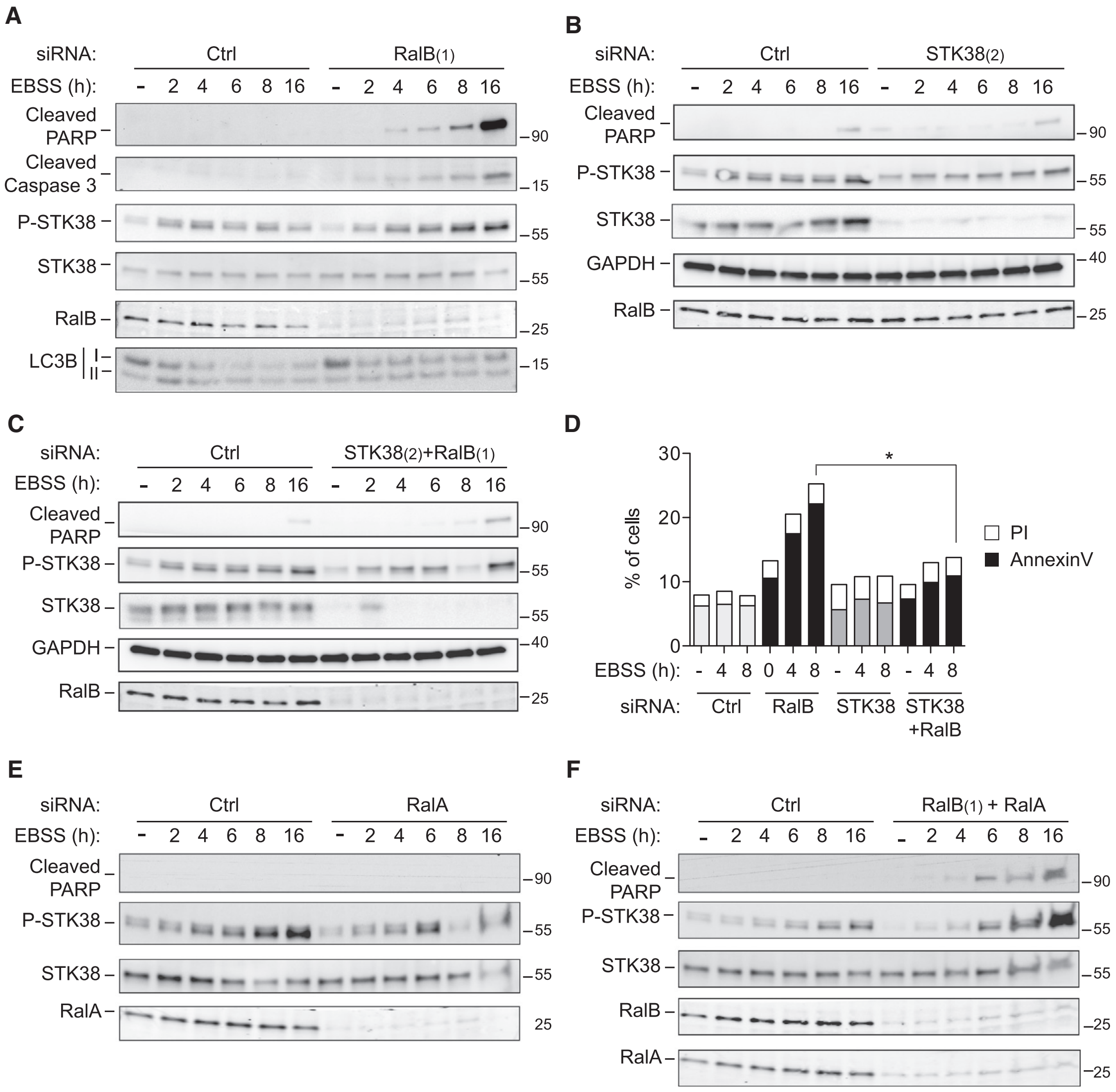

Figure 7. Increased STK38 Activation Drives Apoptosis in RalB-Depleted Cells upon Autophagy Induction

(A–C) HeLa cells were transfected with indicated siRNAs. Seventy-two hours later, cells were treated with EBSS and processed for western blot analysis as shown.

(D) HeLa cells were transfected with indicated siRNAs. Seventy-two hours later, cells were treated with EBSS for the indicated times, subsequently stained for annexin V-FITC and propidium iodide (PI), and analyzed by flow cytometry (n = 3). Histograms represent the percentages of PI-positive cells (empty square) and annexin-V-positive cells (filled square). To determine statistically significant differences, unpaired two-tailed Student's t tests were carried out (*p < 0.05).

(E and F) HeLa cells were transfected with indicated siRNAs. Seventy-two hours later, cells were treated with EBSS and processed for western blot analysis as indicated.

autophagy response (Figure 3). These findings demonstrate that STK38 is required in human and fly cells for autophagy. Most likely, the mechanism by which Trc regulates autophagy in flies is very similar to human STK38, because the autophagic function of Beclin1/ATG6 is conserved between flies and humans [22]. Significantly, expression of constitutively activate Trc in fly larvae was sufficient to induce autophagosome formation (Figure 3), suggesting that Trc kinase activity is not only required but also sufficient to drive autophagosome formation in this setting.

MOB1 has been identified as a key activator of STK38 [12]. Because STK38 activation requires MOB1 binding to the NTR [12] and STK38 is necessary for autophagy induction (Figures 2, 3, and 4), we investigated whether MOB1 is also necessary for autophagy induction. In full support of an autophagic function of STK38, we observed that endogenous MOB1 is, at least to a certain extent, required for autophagy because MOB1 depletion impaired LC3B-II accumulation and autophagy-dependent STK38 activation (Figure 6). Notably, we also mapped the Beclin1 interaction domain to the NTR (residues 1–82) of STK38 (Figure 1), suggesting Beclin1 and MOB1 might compete for the same binding sites. However, neither MOB1 binding to STK38, autophagy induction, nor STK38 kinase activity is required for Beclin1/STK38 complex formation (Figure 1).

Therefore, the initial activation of STK38 by MOB1 binding and STK38/Beclin1 complex formation very likely represent two separate molecular events. Additionally, the MST1/2 kinases can promote STK38 activation in apoptotic and cell-cycle signaling [12]. Intriguingly, MST1/2 kinases are necessary for autophagy in flies and mammals [36, 37]. Consequently, future research into the specific order of events and their regulation is warranted using yet to be developed novel methods and mutants. Considering further that Beclin1 can exist in distinct protein complexes [8, 38] and that our data suggest that Beclin1 can function upstream of STK38 (Figures S3 and S7), future research is also warranted to dissect the relationships between STK38 and different Beclin1-containing complexes to expand our understanding even further. In this regard, as evident from Figure S6B, the apparent dependency of STK38 activation on ULK1 also deserves future investigations in the context of signaling mechanisms involving ULK1 [30, 33]. One possibility is that ULK1 supports the activation of STK38 by MOB1, MST1/2, and/or yet to be identified upstream regulators of STK38 upon autophagy induction. Alternatively, this may involve the direct phosphorylation of STK38 by ULK1 in order to regulate the Exo84/Beclin1/RalB interactions downstream of STK38. To fully appreciate the role of STK38 as an integrator of ULK1 and Beclin signaling in autophagy, it will also be important in the future to define whether and how STK38 acts in feedback and crosstalk mechanisms regulating autophagy.

By studying the regulation of STK38 upon autophagy induction, we further found that STK38 can serve as a link between autophagy and apoptosis. As expected [10], RalB depletion led to an autophagy block (Figure S5). However, this autophagy block was coupled with elevated STK38 phosphorylation (Figure 7), suggesting that RalB, besides its key role in initiating autophagy [10], is also required to fine-tune STK38 activation. This point is important, because in our settings, enhanced STK38 activation resulted in increased apoptosis induction (Figures 7 and S7). Therefore, upon autophagy induction, RalB appears to play a dual role by regulating autophagy and apoptosis. In apoptosis signaling, STK38 functions downstream of RalB, because co-depletion of RalB and STK38 decreased apoptosis induction (Figure 7). In autophagy signaling, future research is warranted to decipher how RalB and STK38 potentially regulate together Exo84/Beclin1 complex formation. In this context, it is notable that our data already suggest that a simple block of autophagosome formation is not sufficient to trigger STK38-dependent apoptosis, because neither Exo84, Beclin1, ULK1 depletion, nor Vps34 inhibition causes STK38 hyperactivation (Figures S5 and S6). Blocking autophagy by Exo84, Beclin1, ULK1 depletion, or 3-MA treatment rather negatively interferes with STK38 activation (Figures S5 and S6), suggesting that activation of STK38 by autophagy induction or RalB depletion might represent two separate molecular events. Therefore, our data propose that RalB-STK38 signaling could potentially serve as a specific switch between cell survival (through autophagy) and cell death (through apoptosis), which when deregulated perhaps can have severe pathophysiological consequences [35]. Consequently, future research into the regulation of the relationship of autophagy and apoptosis by RalB-STK38 signaling is warranted.

Taken together, we identify herein the pro-apoptotic STK38 kinase as a novel positive regulator of autophagy in flies and humans. Moreover, fine-tuning of STK38's activity may represent a critical switch between cell survival (autophagy) and cell death (apoptosis). Therefore, we provide in this manuscript insight into a new regulatory mechanism of cell homeostasis that could possibly be exploited for the development of novel clinical compounds, allowing the modulation of autophagy activity in human diseases.

EXPERIMENTAL PROCEDURES

Further details on experimental procedures (e.g., reagents, plasmids, and siRNAs) are provided in Supplemental Experimental Procedures.

Cell Lines, Transfections, and Autophagy Induction

U2OS (GFP-WIPI-1 and GFP-LC3B) and RPE1 Tet-on HA-STK38-PIF cells were cultured as described [39, 40]. HeLa, HeLa GFP-LC3B, HEK293T, RPE1, RPE1 GFP-LC3B, and HEK-HT were cultured as described in Supplemental Experimental Procedures. siRNA (Eurogentec) transfections were carried out using RNAiMax (Invitrogen) and DNA transfections with Jet Prime reagent (Poly Plus) or Fugene 6 (Promega) following manufacturer's instructions. Autophagy was induced by EBSS or trehalose as defined in Supplemental Experimental Procedures.

Immunoprecipitation Experiments

Beclin1/STK38 co-immunoprecipitations were performed as described [16, 41] using anti-HA 12CA5 or anti-Beclin1 (sc-10087; Santa Cruz) antibodies. HA-Exo84 immunoprecipitations were performed as defined [42].

Western Blot and Densitometry Analysis

After treatments, cells were washed with cold PBS and lysed at 4°C in lysis buffer (20 mM Tris-HCl [pH 8], 150 mM NaCl, 10% glycerol, 1 mM Na_3VO_4, 1% NP40, 1% EDTA, 1 mM β-glycerophosphate, 50 mM NaF, 1 mM DTT, and 1× protease inhibitor [Roche]). Cell lysates were cleared at 13,000 g for 10 min at 4°C. Protein concentrations were determined using the Bio-Rad D_c Protein Assay Kit. Equal amounts of total protein were run on precast gradient SDS-PAGE gels (Bio-Rad). After electrophoresis, proteins were transferred to a 0.2 μm nitrocellulose transfer membrane (Whatman). Membranes were then blotted overnight at 4°C in TBST with 3% BSA with the appropriate primary antibodies. Primary antibodies were detected using appropriate conjugated secondary antibodies and visualized by enhanced chemiluminescence detection (Western Lightning Plus-ECL; PerkinElmer). Densitometric analyses of immunoblots were performed using the Multi Gauge software (FujiFilm).

Flow Cytometry

To determine the number of apoptotic and dead cells, cells were washed, trypsinized, and resuspended in annexin V binding buffer (PharMingen) at a concentration of 1 × 10 E6 cells per ml. Annexin V-APC antibody (5 μl for 100,000 cells) was added and incubated for 15 min in the dark. Before flow cytometry analysis, PI was added to visualize DNA. Data were acquired on a LSRII (BD) or Macsquant (Miltenyi) flow cytometer.

Drosophila Experiments

Strains used were w1118, Cg-Gal4, Act5c-Gal4, Act5c < FRT > CD2 < FRT > Gal4, hsflp-1, UAS-GFP::Atg8a, UAS-mCherry::Atg8a, UAS-GFP, $atg6^{\Delta 1}$ UAS-mRFP, UAS-Trc[S292E], UAS-Trc[S292A+T453A], and UAS-$Trc[IR]^{TRiP.JF02961}$. All crosses and experiments were performed at 25°C as described in detail in Supplemental Experimental Procedures.

Y2H Screens

Y2H screening was performed by Hybrigenics Services (http://www.hybrigenics-services.com) with full-length human STK38 (GenBank: 31377778) and STK38-PIF [15] as baits. See also Supplemental Experimental Procedures.

Reagents, Plasmids, and siRNAs

Anti-phospho-STK38 (Thr444-P) and Mob1 antibodies were previously described [16, 41]. STK38 [41], Exo84-HA [42], and RalB [43] plasmids were

previously described. The sources of the remaining reagents, plasmids, and siRNAs are defined in detail in Supplemental Experimental Procedures.

PI3P Formation Measurements, Immunofluorescence, Time-Lapse Microscopy, and Image Analysis

PI3P formation was measured as described [44]. LC3B and ATG12 immunofluorescence staining and quantifications as well as time-lapse microscopy are described in detail in Supplemental Experimental Procedures.

Statistical Analysis

Unless otherwise indicated, unpaired two-tailed Student's t tests were carried out. Quantitative data of the indicated number of independent experiments ("n =" in figure legends) are expressed as means ± SEM. For statistical analysis of *Drosophila* data, one-tail unpaired Student's t test and one-way ANOVA followed by post hoc Bonferroni's multiple comparison test were performed as described in the respective figure legends. For all tests, differences were considered statistically significant when p values were below 0.05 (*), 0.01 (**), or 0.001 (***). In the figures, p values are indicated as $^{*}p < 0.05$, $^{**}p < 0.01$, and $^{***}p < 0.001$.

SUPPLEMENTAL INFORMATION

Supplemental Information includes Supplemental Discussion, Supplemental Experimental Procedures, seven figures, and two movies and can be found with this article online at http://dx.doi.org/10.1016/j.cub.2015.08.031.

AUTHOR CONTRIBUTIONS

P.C., M.F., A.H., and J.C. directed the project. C.J. performed all experiments with the following exceptions: N.P. performed some experiments shown in Figures 1 and 4; L.H. and V.G. performed some experiments shown in Figures 1 and S7; R.P. and C.G.-P. performed experiments shown in Figures 3, S3, and S7; A.B. performed some experiments shown in Figure S2; C.B. performed some experiments shown in Figure S4; B.M. supported some biochemical experiments supervised by C.J., J.C., and I.C.; I.C. further supported the analysis and interpretation of experiments performed by B.M.; and A.H. designed Figure 5D. Tet-on cell lines were generated by L.H. and A.H. C.J., N.P., V.G., R.P., C.G.-P., M.F., P.C., A.H., and J.C. interpreted the data. C.J. and A.H. wrote the manuscript. All authors contributed with discussion and edited the manuscript.

ACKNOWLEDGMENTS

We thank I. Bjedov, J. Lisztwan, O. Joffre, and S. Manenti for their support and critical feedback. We thank T. Yoshimori and Q. Zhong for kindly providing plasmids pftLC3, Beclin1-HA, and Beclin1-DsRed through Addgene. We thank also T. Proikas-Cezanne for her kind gift of UO2S-GFP-WIPI-1 and GFP-LC3B cells and J. Murray and H. Stenmark for giving us Vps34-RFP and FYVE peptide, respectively. We thank E. Baehrecke, T. Neufeld, T.E. Rusten, and I. Nezis for fly stocks and reagents. RPE1 GFP-LC3B cells were kindly provided by A. Lopez Rivas. Further, we thank L. Sengmanivong at the Nikon Imaging Center (Institut Curie Centre National de la Recherche Scientifique), the Necker Institute Imaging Facility with the fondation Imagine for technical assistance with microscopy, and HYBRIGENICS services for their advice in Y2H screens. This work was supported by grants from the Institut National de la Santé et de la Recherche Médicale (INSERM), the Institut Curie, the Ligue Nationale Contre le Cancer, the Association pour la Recherche sur le Cancer (ARC), the Institut National du Cancer (INCa), AICR (11-0634) awarded to A.H., and by a New Investigator Research Grant from the Medical Research Council (G1002186) and a grant from the National Centre for the 3Rs (NC/L000199/1) awarded to M.F. A.H. is a Wellcome Trust Research Career Development fellow (090090/Z/09/Z). N.D. is a recipient of Fondation pour la Recherche Médicale fellowship.

Received: December 17, 2014
Revised: July 9, 2015
Accepted: August 13, 2015
Published: September 17, 2015

REFERENCES

1. Mizushima, N., and Komatsu, M. (2011). Autophagy: renovation of cells and tissues. Cell *147*, 728–741.
2. Boya, P., Reggiori, F., and Codogno, P. (2013). Emerging regulation and functions of autophagy. Nat. Cell Biol. *15*, 713–720.
3. Ravikumar, B., Sarkar, S., Davies, J.E., Futter, M., Garcia-Arencibia, M., Green-Thompson, Z.W., Jimenez-Sanchez, M., Korolchuk, V.I., Lichtenberg, M., Luo, S., et al. (2010). Regulation of mammalian autophagy in physiology and pathophysiology. Physiol. Rev. *90*, 1383–1435.
4. Choi, A.M.K., Ryter, S.W., and Levine, B. (2013). Autophagy in human health and disease. N. Engl. J. Med. *368*, 651–662.
5. Behrends, C., Sowa, M.E., Gygi, S.P., and Harper, J.W. (2010). Network organization of the human autophagy system. Nature *466*, 68–76.
6. Dengjel, J., Høyer-Hansen, M., Nielsen, M.O., Eisenberg, T., Harder, L.M., Schandorff, S., Farkas, T., Kirkegaard, T., Becker, A.C., Schroeder, S., et al. (2012). Identification of autophagosome-associated proteins and regulators by quantitative proteomic analysis and genetic screens. Mol. Cell. Proteomics *11*, M111.014035.
7. Orvedahl, A., Sumpter, R., Jr., Xiao, G., Ng, A., Zou, Z., Tang, Y., Narimatsu, M., Gilpin, C., Sun, Q., Roth, M., et al. (2011). Image-based genome-wide siRNA screen identifies selective autophagy factors. Nature *480*, 113–117.
8. Wirth, M., Joachim, J., and Tooze, S.A. (2013). Autophagosome formation–the role of ULK1 and Beclin1-PI3KC3 complexes in setting the stage. Semin. Cancer Biol. *23*, 301–309.
9. Lamb, C.A., Yoshimori, T., and Tooze, S.A. (2013). The autophagosome: origins unknown, biogenesis complex. Nat. Rev. Mol. Cell Biol. *14*, 759–774.
10. Bodemann, B.O., Orvedahl, A., Cheng, T., Ram, R.R., Ou, Y.-H., Formstecher, E., Maiti, M., Hazelett, C.C., Wauson, E.M., Balakireva, M., et al. (2011). RalB and the exocyst mediate the cellular starvation response by direct activation of autophagosome assembly. Cell *144*, 253–267.
11. Hergovich, A., Stegert, M.R., Schmitz, D., and Hemmings, B.A. (2006). NDR kinases regulate essential cell processes from yeast to humans. Nat. Rev. Mol. Cell Biol. *7*, 253–264.
12. Hergovich, A. (2013). Regulation and functions of mammalian LATS/NDR kinases: looking beyond canonical Hippo signalling. Cell Biosci. *3*, 32.
13. Emoto, K., Parrish, J.Z., Jan, L.Y., and Jan, Y.-N. (2006). The tumour suppressor Hippo acts with the NDR kinases in dendritic tiling and maintenance. Nature *443*, 210–213.
14. He, Y., Fang, X., Emoto, K., Jan, Y.-N., and Adler, P.N. (2005). The tricornered Ser/Thr protein kinase is regulated by phosphorylation and interacts with furry during Drosophila wing hair development. Mol. Biol. Cell *16*, 689–700.
15. Cook, D., Hoa, L.Y., Gomez, V., Gomez, M., and Hergovich, A. (2014). Constitutively active NDR1-PIF kinase functions independent of MST1 and hMOB1 signalling. Cell. Signal. *26*, 1657–1667.
16. Hergovich, A., Kohler, R.S., Schmitz, D., Vichalkovski, A., Cornils, H., and Hemmings, B.A. (2009). The MST1 and hMOB1 tumor suppressors control human centrosome duplication by regulating NDR kinase phosphorylation. Curr. Biol. *19*, 1692–1702.
17. Sarkar, S., Davies, J.E., Huang, Z., Tunnacliffe, A., and Rubinsztein, D.C. (2007). Trehalose, a novel mTOR-independent autophagy enhancer, accelerates the clearance of mutant huntingtin and alpha-synuclein. J. Biol. Chem. *282*, 5641–5652.
18. Tallóczy, Z., Jiang, W., Virgin, H.W., 4th, Leib, D.A., Scheuner, D., Kaufman, R.J., Eskelinen, E.-L., and Levine, B. (2002). Regulation of starvation- and virus-induced autophagy by the eIF2alpha kinase signaling pathway. Proc. Natl. Acad. Sci. USA *99*, 190–195.
19. Klionsky, D.J., Abdalla, F.C., Abeliovich, H., Abraham, R.T., Acevedo-Arozena, A., Adeli, K., Agholme, L., Agnello, M., Agostinis, P., Aguirre-Ghiso, J.A., et al. (2012). Guidelines for the use and interpretation of assays for monitoring autophagy. Autophagy *8*, 445–544.

20. Bjørkøy, G., Lamark, T., and Johansen, T. (2006). p62/SQSTM1: a missing link between protein aggregates and the autophagy machinery. Autophagy *2*, 138–139.

21. Geng, W., He, B., Wang, M., and Adler, P.N. (2000). The tricornered gene, which is required for the integrity of epidermal cell extensions, encodes the Drosophila nuclear DBF2-related kinase. Genetics *156*, 1817–1828.

22. Juhász, G., Hill, J.H., Yan, Y., Sass, M., Baehrecke, E.H., Backer, J.M., and Neufeld, T.P. (2008). The class III PI(3)K Vps34 promotes autophagy and endocytosis but not TOR signaling in Drosophila. J. Cell Biol. *181*, 655–666.

23. Shravage, B.V., Hill, J.H., Powers, C.M., Wu, L., and Baehrecke, E.H. (2013). Atg6 is required for multiple vesicle trafficking pathways and hematopoiesis in Drosophila. Development *140*, 1321–1329.

24. Schink, K.O., Raiborg, C., and Stenmark, H. (2013). Phosphatidylinositol 3-phosphate, a lipid that regulates membrane dynamics, protein sorting and cell signalling. BioEssays *35*, 900–912.

25. Kimura, S., Noda, T., and Yoshimori, T. (2007). Dissection of the autophagosome maturation process by a novel reporter protein, tandem fluorescent-tagged LC3. Autophagy *3*, 452–460.

26. Rifki, O.F., Bodemann, B.O., Battiprolu, P.K., White, M.A., and Hill, J.A. (2013). RalGDS-dependent cardiomyocyte autophagy is required for load-induced ventricular hypertrophy. J. Mol. Cell. Cardiol. *59*, 128–138.

27. Simicek, M., Lievens, S., Laga, M., Guzenko, D., Aushev, V.N., Kalev, P., Baietti, M.F., Strelkov, S.V., Gevaert, K., Tavernier, J., and Sablina, A.A. (2013). The deubiquitylase USP33 discriminates between RALB functions in autophagy and innate immune response. Nat. Cell Biol. *15*, 1220–1230.

28. Hergovich, A. (2011). MOB control: reviewing a conserved family of kinase regulators. Cell. Signal. *23*, 1433–1440.

29. Hergovich, A., Lamla, S., Nigg, E.A., and Hemmings, B.A. (2007). Centrosome-associated NDR kinase regulates centrosome duplication. Mol. Cell *25*, 625–634.

30. Russell, R.C., Tian, Y., Yuan, H., Park, H.W., Chang, Y.-Y., Kim, J., Kim, H., Neufeld, T.P., Dillin, A., and Guan, K.-L. (2013). ULK1 induces autophagy by phosphorylating Beclin-1 and activating VPS34 lipid kinase. Nat. Cell Biol. *15*, 741–750.

31. Nazio, F., Strappazzon, F., Antonioli, M., Bielli, P., Cianfanelli, V., Bordi, M., Gretzmeier, C., Dengjel, J., Piacentini, M., Fimia, G.M., and Cecconi, F. (2013). mTOR inhibits autophagy by controlling ULK1 ubiquitylation, self-association and function through AMBRA1 and TRAF6. Nat. Cell Biol. *15*, 406–416.

32. Burnett, P.E., Barrow, R.K., Cohen, N.A., Snyder, S.H., and Sabatini, D.M. (1998). RAFT1 phosphorylation of the translational regulators p70 S6 kinase and 4E-BP1. Proc. Natl. Acad. Sci. USA *95*, 1432–1437.

33. Kim, J., Kundu, M., Viollet, B., and Guan, K.-L. (2011). AMPK and mTOR regulate autophagy through direct phosphorylation of Ulk1. Nat. Cell Biol. *13*, 132–141.

34. Vichalkovski, A., Gresko, E., Cornils, H., Hergovich, A., Schmitz, D., and Hemmings, B.A. (2008). NDR kinase is activated by RASSF1A/MST1 in response to Fas receptor stimulation and promotes apoptosis. Curr. Biol. *18*, 1889–1895.

35. Mariño, G., Niso-Santano, M., Baehrecke, E.H., and Kroemer, G. (2014). Self-consumption: the interplay of autophagy and apoptosis. Nat. Rev. Mol. Cell Biol. *15*, 81–94.

36. Napoletano, F., Occhi, S., Calamita, P., Volpi, V., Blanc, E., Charroux, B., Royet, J., and Fanto, M. (2011). Polyglutamine Atrophin provokes neurodegeneration in Drosophila by repressing fat. EMBO J. *30*, 945–958.

37. Wilkinson, D.S., Jariwala, J.S., Anderson, E., Mitra, K., Meisenhelder, J., Chang, J.T., Ideker, T., Hunter, T., Nizet, V., Dillin, A., and Hansen, M. (2015). Phosphorylation of LC3 by the Hippo kinases STK3/STK4 is essential for autophagy. Mol. Cell *57*, 55–68.

38. Levine, B., Liu, R., Dong, X., and Zhong, Q. (2015). Beclin orthologs: integrative hubs of cell signaling, membrane trafficking, and physiology. Trends Cell Biol. *25*, 533–544.

39. Proikas-Cezanne, T., Ruckerbauer, S., Stierhof, Y.-D., Berg, C., and Nordheim, A. (2007). Human WIPI-1 puncta-formation: a novel assay to assess mammalian autophagy. FEBS Lett. *581*, 3396–3404.

40. Gomez-Martinez, M., Schmitz, D., and Hergovich, A. (2013). Generation of stable human cell lines with tetracycline-inducible (Tet-on) shRNA or cDNA expression. J. Vis. Exp. *2013*, e50171.

41. Hergovich, A., Bichsel, S.J., and Hemmings, B.A. (2005). Human NDR kinases are rapidly activated by MOB proteins through recruitment to the plasma membrane and phosphorylation. Mol. Cell. Biol. *25*, 8259–8272.

42. Parrini, M.C., Sadou-Dubourgnoux, A., Aoki, K., Kunida, K., Biondini, M., Hatzoglou, A., Poullet, P., Formstecher, E., Yeaman, C., Matsuda, M., et al. (2011). SH3BP1, an exocyst-associated RhoGAP, inactivates Rac1 at the front to drive cell motility. Mol. Cell *42*, 650–661.

43. Cascone, I., Selimoglu, R., Ozdemir, C., Del Nery, E., Yeaman, C., White, M., and Camonis, J. (2008). Distinct roles of RalA and RalB in the progression of cytokinesis are supported by distinct RalGEFs. EMBO J. *27*, 2375–2387.

44. Khaldoun, S.A., Emond-Boisjoly, M.-A., Chateau, D., Carrière, V., Lacasa, M., Rousset, M., Demignot, S., and Morel, E. (2014). Autophagosomes contribute to intracellular lipid distribution in enterocytes. Mol. Biol. Cell *25*, 118–132.

Current Biology

Supplemental Information

The Pro-apoptotic STK38 Kinase Is a New Beclin1 Partner Positively Regulating Autophagy

Carine Joffre, Nicolas Dupont, Lily Hoa, Valenti Gomez, Raul Pardo, Catarina Gonçalves-Pimentel, Pauline Achard, Audrey Bettoun, Brigitte Meunier, Chantal Bauvy, Ilaria Cascone, Patrice Codogno, Manolis Fanto, Alexander Hergovich, and Jacques Camonis

Figure S1

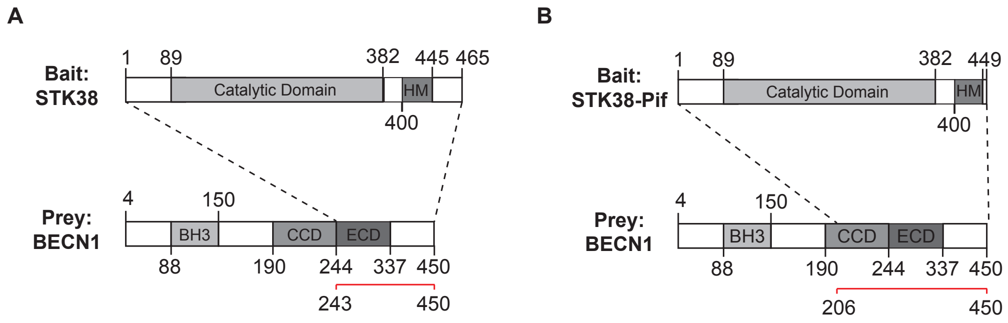

Yeast two hybrid interactions between STK38/STK38-PIF and Beclin1 (in support of Figure 1).

Primary structure of human STK38 (**A**), STK-PIF (**B**) and Beclin1 illustrating yeast two-hybrid results. Domains required for interactions between STK38 and Beclin1 are indicated. CCD: coiled-coil domain; ECD: evolutionarily conserved domain, HM: hydrophobic motif.

Figure S2

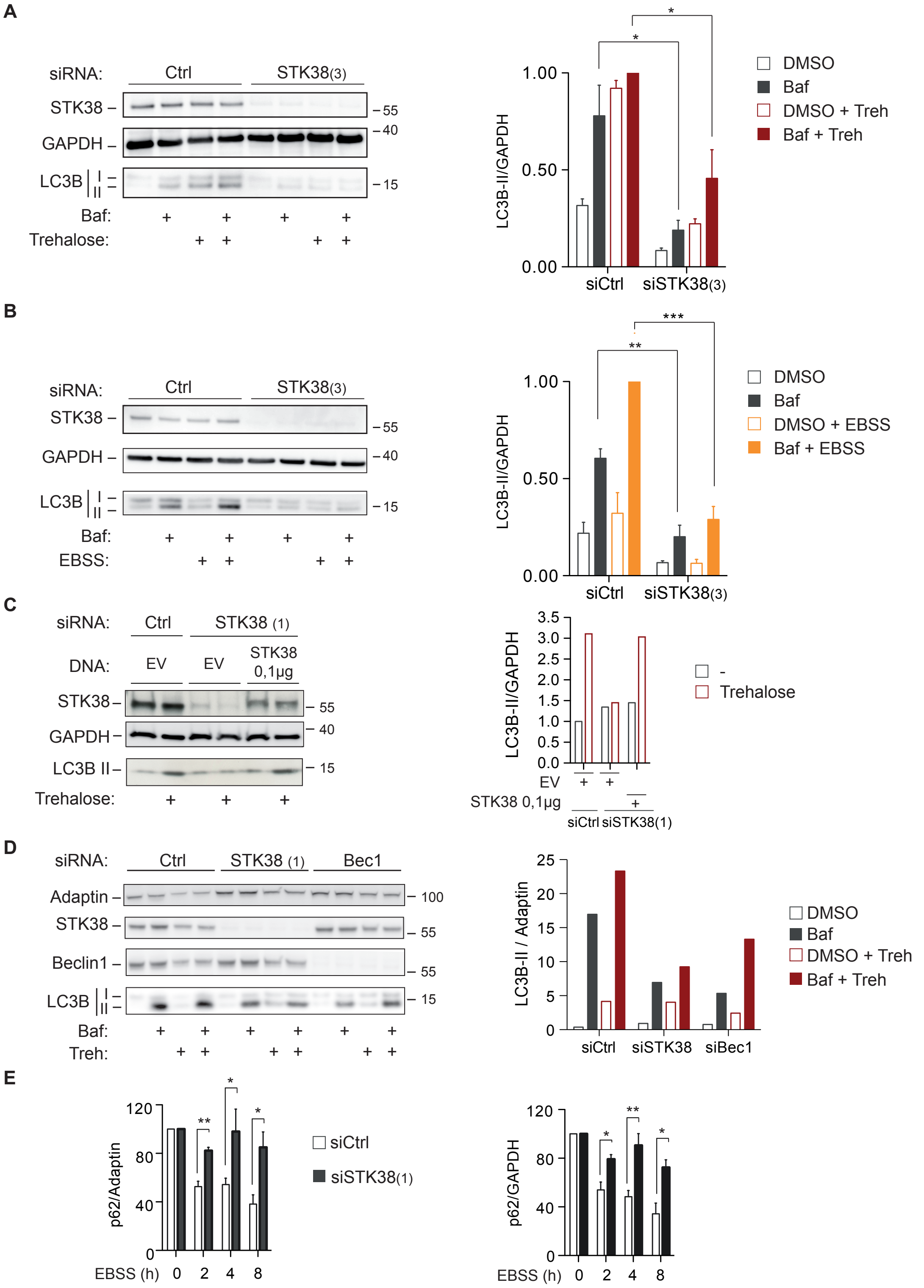
A
siRNA:
Ctrl
STK38(3)
STK38
GAPDH
LC3B
I
II
55
40
15
Baf:
Trehalose:
LC3B-II/GAPDH
1.00
0.50
0.00
siCtrl
siSTK38(3)
*
DMSO
Baf
DMSO + Treh
Baf + Treh
B
siRNA:
Ctrl
STK38(3)
STK38
GAPDH
LC3B
55
40
15
Baf:
EBSS:
LC3B-II/GAPDH
**
***
DMSO
Baf
DMSO + EBSS
Baf + EBSS
C
siRNA:
Ctrl
STK38 (1)
DNA:
EV
STK38 0,1µg
STK38
GAPDH
LC3B II
Trehalose:
LC3B-II/GAPDH
3.5
3.0
2.5
2.0
1.5
1.0
0.5
0.0
EV
STK38 0,1µg
siCtrl
siSTK38(1)
-
Trehalose
D
siRNA:
Ctrl
STK38 (1)
Bec1
Adaptin
STK38
Beclin1
LC3B
100
55
15
Baf:
Treh:
LC3B-II / Adaptin
25
20
15
10
5
0
siCtrl
siSTK38
siBec1
DMSO
Baf
DMSO + Treh
Baf + Treh
E
p62/Adaptin
120
80
40
0
EBSS (h)
0
2
4
8
siCtrl
siSTK38(1)
p62/GAPDH

Data in support of Figure 2.

(A,B) STK38 depletion impairs the dynamics of LC3B-II levels upon autophagy induction. HeLa cells were transfected with indicated siRNAs. 72 h later, cells were incubated with Trehalose for 16 h (A) or EBSS for 4 h (B) with or without BafilomycinA1 (Baf) for 4 h, followed by immunoblotting using indicated antibodies (left panels). Histograms represent LC3B-II/GAPDH ratios obtained by densitometric analysis (n=3 +/- sem) of Western blots (right panels).

(C) RNAi-resistant STK38(wt) restores autophagy in STK38-depleted cells. STK38-depleted HeLa cells (48 h) were transfected with RNAi-resistant STK38 wild-type. 24 h later, cells were treated with Trehalose and LC3B-II levels were assessed by Western blots (left panel). Histogram shows LC3B-II/GAPDH ratios obtained by densitometric analysis of Western blots (right panel).

(D) STK38 is important for autophagy induction similar to Beclin1. HeLa cells were transfected with indicated siRNAs. 72 h later cells were treated with Trehalose for 16 h with or without Bafilomycin for 2 h, followed by processing for immunoblotting using indicated antibodies (left panel). Histograms show LC3B-II/Adaptin ratios obtained by densitometric analysis (right panel).

(E) STK38 depletion impairs the dynamics of p62 levels upon autophagy induction. HeLa cells were transfected with indicated siRNAs. 72 h later cells were subjected to EBSS treatment as indicated. p62 level was assessed by Western blotting (left panel). Histograms show p62/Adaptin and p62/GAPDH ratios obtained by densitometric analysis ($n \geq 3$ +/- sem) of Western blots (right panel). To determine statistically significant differences unpaired two-tailed Student's t-tests were carried out (* $p<0.05$, ** $p<0.01$, *** $p<0.001$).

Figure S3

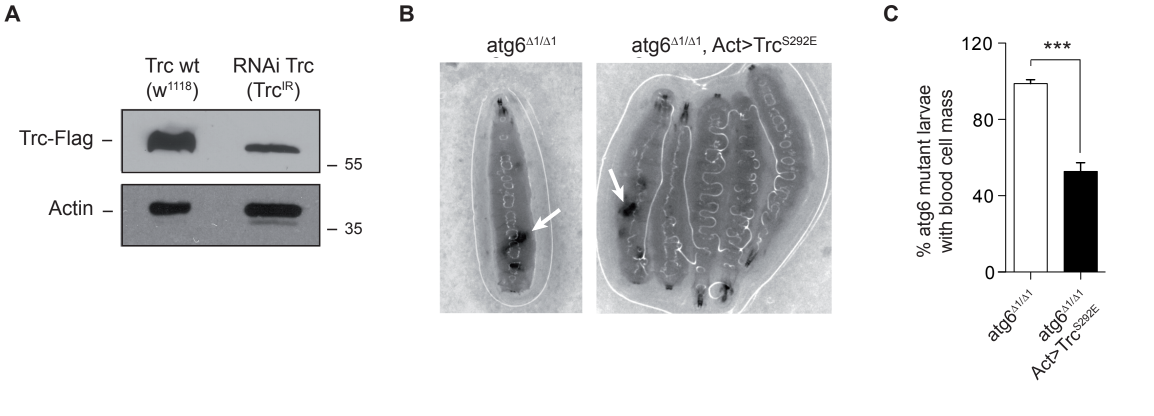

Data in support of Figure 3.

(A) Validation of Trc depletion by RNAi Trc. *Drosophila* heads with the indicated genotypes were processed for immunoblotting with the indicated antibodies. Noteworthy, exogenous Flag-tagged wild-type Trc was depleted in flies expressing the RNAi transgene directed against Trc.

(B,C) Activated Trc (fly STK38) can partially compensate for *atg6* loss-of-function. (B) Brightfield images of third instar fly larvae of an *atg6* mutant (left panel) and *atg6* mutants expressing activated Trc^{S292E} (right panel). Melanotic blood cell masses are visible as black masses (indicated by arrows). (C) Histograms show the quantification of larvae displaying blood cell masses (n=70). To determine statistically significant differences unpaired two-tailed Student's t-tests were carried out (*** $p<0.001$).

Figure S4

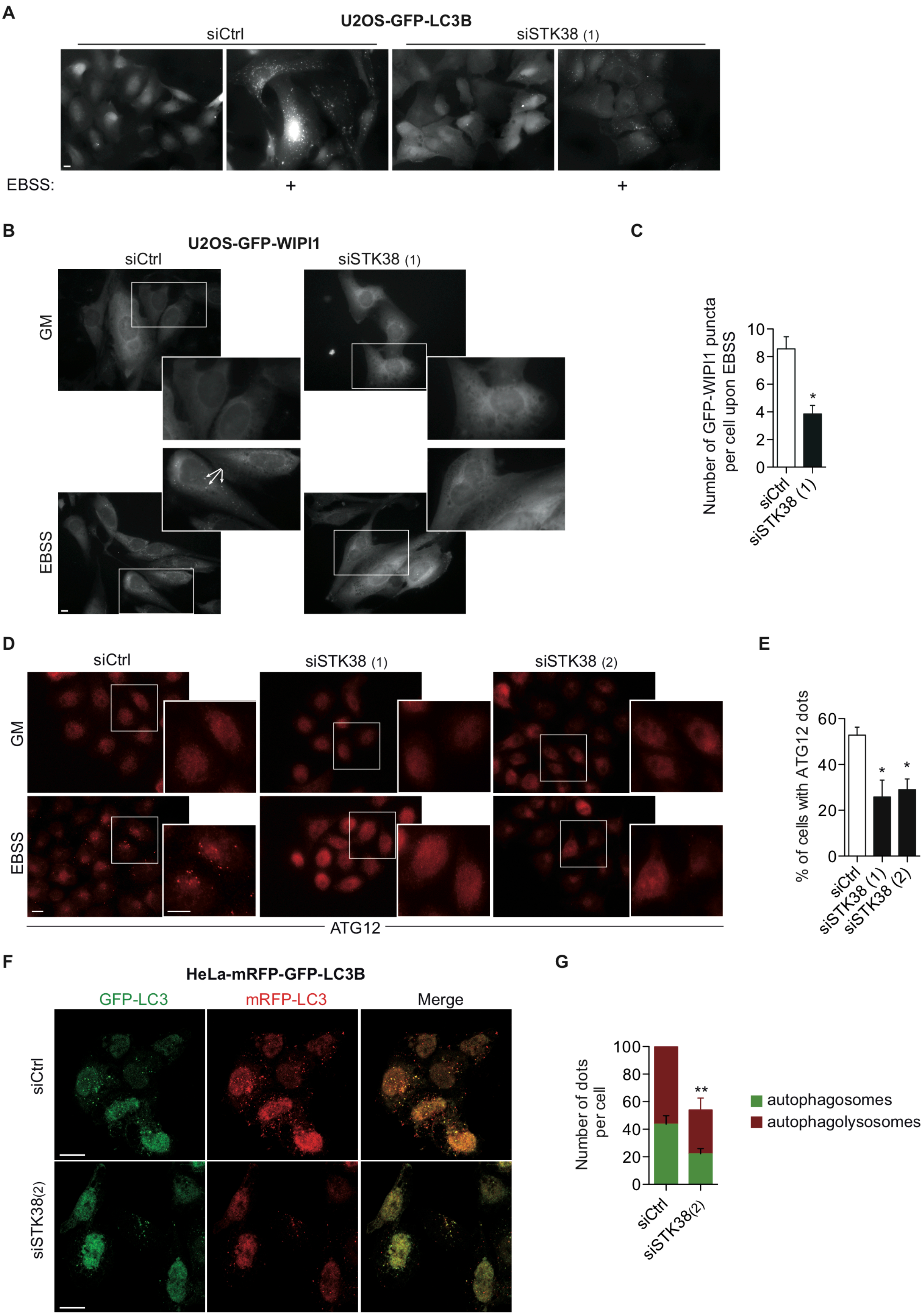

A
U2OS-GFP-LC3B
siCtrl
siSTK38 (1)
EBSS:
+
+
B
U2OS-GFP-WIPI1
siCtrl
siSTK38 (1)
GM
EBSS
C
Number of GFP-WIPI1 puncta per cell upon EBSS
10
8
6
4
2
0
*
siCtrl
siSTK38 (1)
D
siCtrl
siSTK38 (1)
siSTK38 (2)
GM
EBSS
ATG12
E
% of cells with ATG12 dots
60
40
20
0
*
*
siCtrl
siSTK38 (1)
siSTK38 (2)
F
HeLa-mRFP-GFP-LC3B
GFP-LC3
mRFP-LC3
Merge
siCtrl
siSTK38(2)
G
Number of dots per cell
100
80
60
40
20
0
**
siCtrl
siSTK38(2)
autophagosomes
autophagolysosomes

STK38 plays a role in early autophagic events (in support of Figure 4).

(A) U2OS cells stably expressing GFP-LC3B were transfected with indicated siRNAs. 72 h later cells were treated with EBSS for 2 h and fixed. Representative pictures are shown.

(B) U2OS cells stably expressing GFP-WIPI1 were transfected with indicated siRNAs. 72 h later cells were treated with EBSS for 2 h and fixed. Representative pictures are shown.

(C) Quantifications for experiments in (B). The histogram represents the number of GFP-WIPI1 puncta per cell upon EBSS treatment, quantifications performed with Image J software (n=3 +/- sem).

(D) HeLa cells were transfected with indicated siRNAs. 72 h later, cells were treated with EBSS for 2 h and stained for ATG12. Representative confocal pictures are shown. Scale bar: 10μM

(E) Quantification of experiments shown in (D). Histograms represent the percentage of cells displaying at least one ATG12 dot (n=3 +/- sem).

(F) HeLa-mRFP-GFP-LC3B cells were transfected with indicated siRNAs. 72 h after plating, cells were treated with EBSS for 2h and processed for immunofluorescence. Representative images are shown. Scale bar: 10 μM

(G) Quantification of experiments shown in (F). Histograms display the percentage of autophagosomes (yellow dots in F) and autophagolysosomes (red dots in F) per cell (n=3 +/- sem).

Figure S5

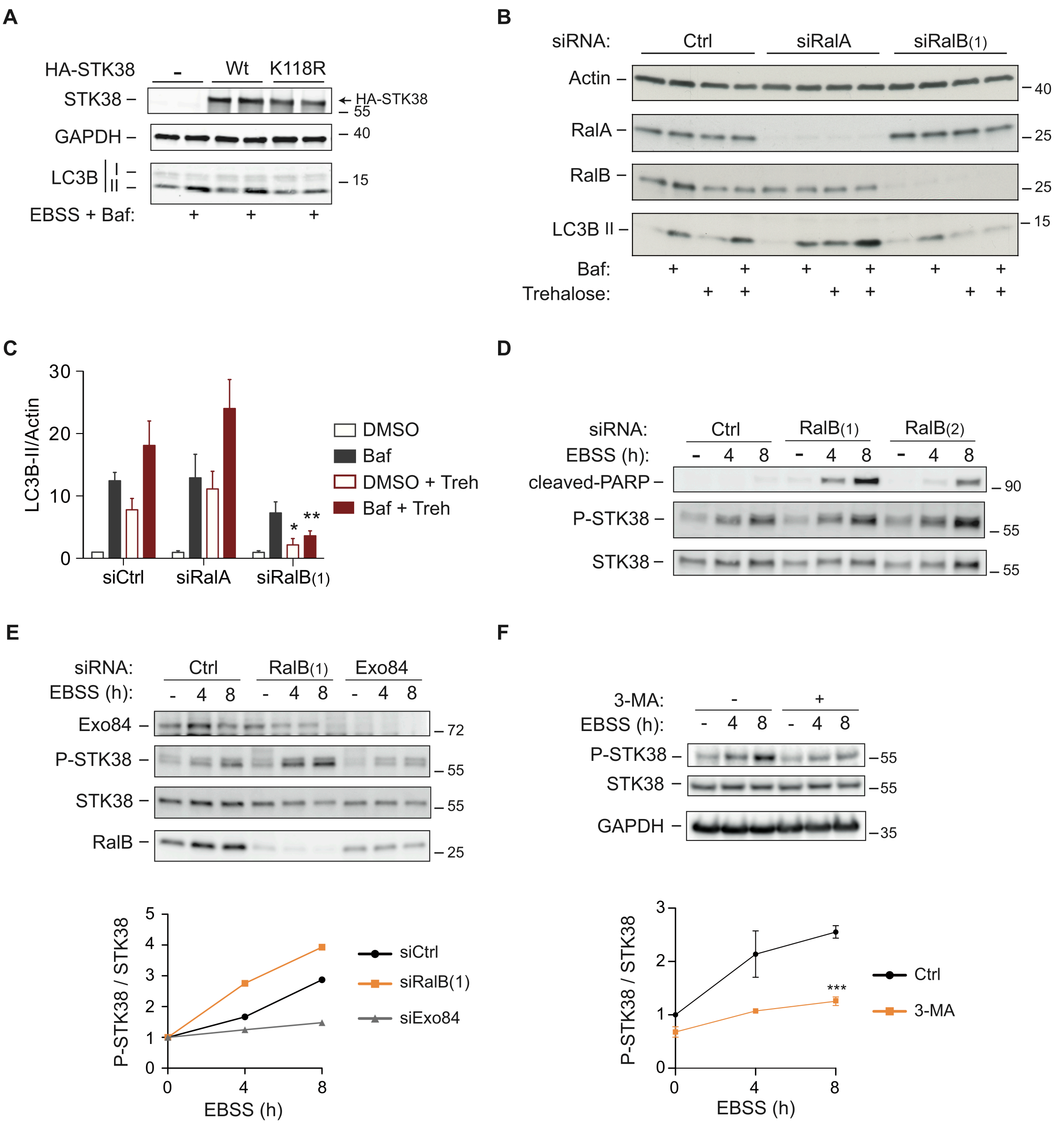

Data in support of Figure 6.

(A) Dominant negative kinase-dead STK38 interferes with autophagy induction. HeLa cells were transfected with an empty vector (-) or with a plasmid expressing either an HA tagged Wt STK38 or a kinase dead STK38 form. 24 h later cells were treated with EBSS for 2 h, followed by processing for immunoblotting using indicated antibodies. Wt, wild type; K118R, kinase-dead.

(B,C,D) RalB depletion blocks autophagy, but promotes apoptosis and STK38 activation. (B) HeLa cells were transfected with indicated siRNAs. 48 h later cells were treated with Trehalose for 16 h, followed by processing for immunoblotting using indicated antibodies. (C) Quantifications for experiments in (B). The histogram shows the LC3B-II/Actin ratios

obtained by densitometric analysis of Western blots (n=3 +/- sem). (D) HeLa cells were transfected with indicated siRNAs. 72 h later cells were treated with EBSS for the indicated times followed by processing for immunoblotting using indicated antibodies.

(E,F) Exo84 depletion and 3-MA treatment block EBSS-induced STK38 activation. (E) HeLa cells were transfected with indicated siRNAs. 72 h later cells were treated with EBSS for the indicated times followed by processing for immunoblotting using indicated antibodies. (F) HeLa cells were treated with 3-methyladenine (10mM) and stimulated with EBSS as indicated followed by processing for immunoblotting.

Figure S6

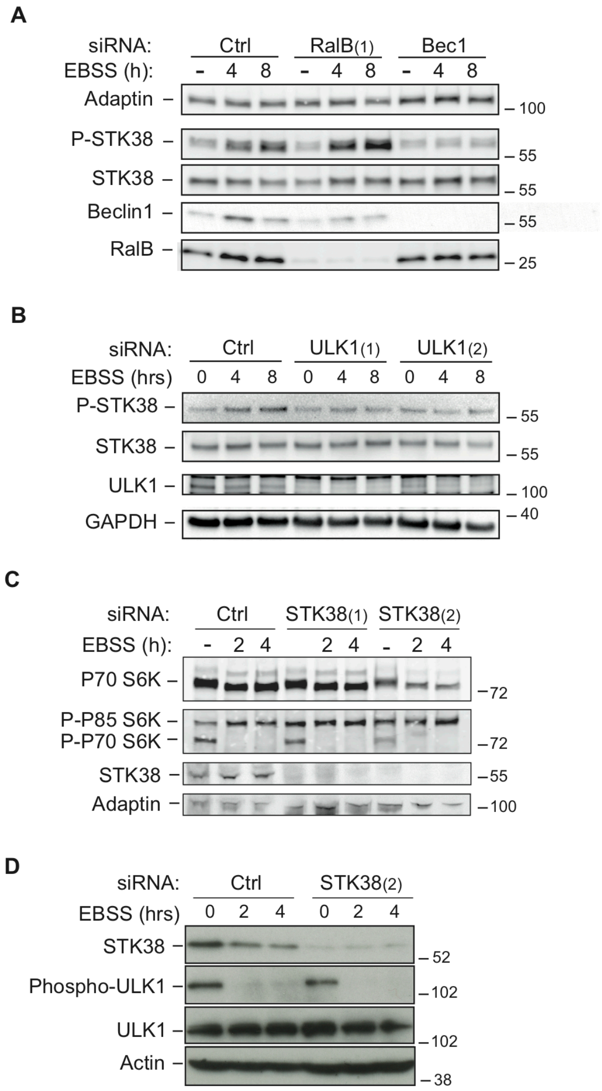

Beclin1 and ULK1 depletion impairs EBSS-induced STK38 activation, while STK38 depletion does not affect the suppression of mTOR-mediated phosphorylation of p70(S6K) on S389 and ULK1 on S757 upon nutrient deprivation (in support of Figure 6)
(A,B) HeLa cells were transfected with indicated siRNAs. 72 h later cells were treated with EBSS for the indicated times followed by processing for immunoblotting using indicated antibodies. Noteworthy, the EBSS-induced phosphorylation of STK38 is diminished in Beclin1 (A) and ULK1 (B) depleted cells.
(C,D) HeLa cells were transfected with indicated siRNAs. 72 h later cells were treated with EBSS for the indicated times followed by processing for immunoblotting using indicated antibodies. p70(S6K) and ULK1 phosphorylation by mTOR is decreased in control and STK38-depleted cells upon EBSS treatment, suggesting that STK38 is dispensable for the suppression of mTOR upon nutrient deprivation.

Figure S7

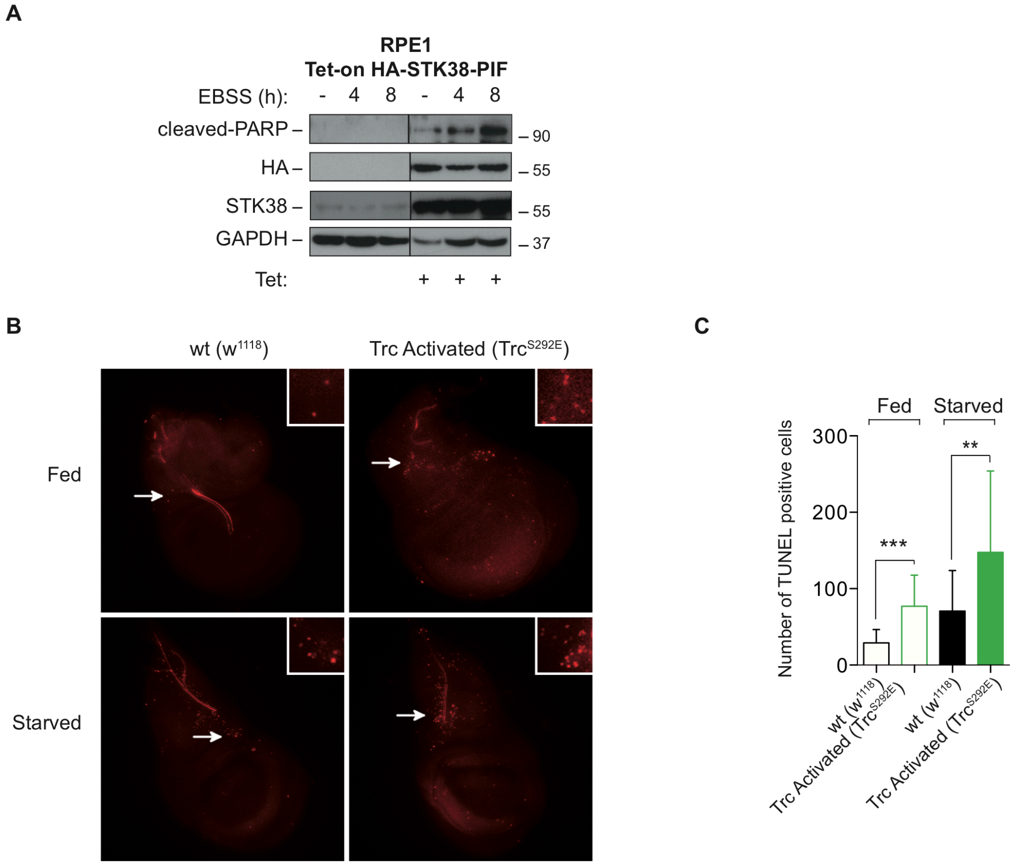

Data in support of Figure 7.

(A) Increased STK38 activity is sufficient to increase EBSS-induced apoptosis. RPE1 Tet-on HA-STK38-PIF cells were incubated with (+) or without tetracycline (2 µg/ml) for 24 h, before EBSS treatment as indicated and subsequent processing for immunoblotting using indicated antibodies. The Western blots shown were assembled from the same exposure.

(B,C) Activated Trc can drive increased apoptosis in fly larvae. (B) Fluorescent images of TUNEL staining of wing discs from fed or starved fly larvae expressing either no Gal4 driver (control Trc wt) or activated Trc^{S292E}. Magnifications of areas indicated by white arrows are shown as insets. Apoptotic cells are shown in red. (C) Histograms show the quantification of the number of TUNEL positive cells detected in each condition (n=20). To determine statistically significant differences unpaired two-tailed Student's t-tests were carried out (** P<0.01, *** p<0.001).

Supplemental Discussion

As a protein kinase [S1], STK38 represents a potentially interesting drug target to modulate autophagy activity in human diseases associated with deregulated autophagy [S2]. In the case of neurodegenerative disorders with low levels of autophagy, autophagy activity would need to be increased. Similarly, in infectious diseases autophagy would preferably also be increased to remove pathogens efficiently. In contrast, inhibition of autophagy in established tumors represents a good therapeutic option to overcome chemoresistance and decrease tumor growth [S3], although decreased autophagic activity can promote tumorigenesis [S4]. Therefore, it would be ideal to have agents that either increase or decrease autophagy activity depending on the medical condition of patients. In this context, STK38 inhibitors would require stringent evaluation as STK38 has other cellular functions [S1] besides the role in autophagy described here. In particular, future studies will need to address whether inhibition of STK38 could result in blood borne diseases in humans, since STK38 knock-out mice are prone to develop T cell lymphoma and myeloproliferative disease [S5]. Intriguingly, defective autophagy in hematopoietic stem cells leads to similar immune system diseases [S6], indicating that STK38 null mice might develop lymphoma and/or myeloproliferative disease due to defective autophagy. Consequently, it would be worth re-examining these animals in this respect.

Supplemental Experimental Procedures

Cell lines and transfections

HeLa, HeLa GFP-LC3B, HEK293T, RPE1, and RPE1 GFP-LC3B cells were cultured in RPMI with Glutamax (Gibco), DMEM (Gibco) with 15mM of Hepes (Sigma), or DMEM-F12 with Glutamax (Gibco), respectively. HEK-HT cells were cultured in DMEM medium with 1% L-glutamine (Gibco) and hygromycin 100μg/mL and neomycin 400μg/mL (Invivogen). All media were supplemented with 10% fetal bovine serum (FBS, Gibco) unless otherwise indicated. Cells were cultured at 37°C and 5% CO_2 in a humidified chamber except for U2OS that were cultured at 37°C and 10% CO_2.

Autophagy induction

Cells are plated 2 days (or 3 days for siRNA experiment) before autophagy induction to ensure consistent confluency (below 70%) for experiments. For EBSS-induced autophagy, medium was aspirated, and cells quickly washed once with growth medium (GM) or EBSS (24010-43, Gibco), followed by incubation with GM or EBSS as indicated. For trehalose-induced autophagy, medium was aspirated and immediately replaced with GM containing 100mM trehalose (T0167, Sigma).

Drosophila Experiments

Strains used were: *w1118*, *Cg-Gal4*, *Act5c-Gal4, Act5c<FRT>CD2<FRT>Gal4, hsflp-1, UAS-GFP::Atg8a*, *UAS-mCherry::Atg8a, UAS-GFP, atg6*$^{\Delta 1}$ *UAS-mRFP*, *UAS-Trc[S292E]*, *UAS-Trc[S292A+T453A]*, *UAS-Trc[IR]*$^{TRiP.JF02961}$. All crosses and experiments were performed at 25°C. Third instar larvae were starved 6h in PBS + 20% sucrose to induce

autophagy. Fat bodies were dissected, mounted in Vectashield (Vectorlabs) and readily imaged on a confocal microscope (Olympus, Leica or Zeiss). For the GFP cleavage assay fat bodies from 10 larvae from each genotype and conditions were dissected and snap frozen before grinding in Laemmli buffer. GFP was detected by immunoblotting with a mouse monoclonal antibody (Roche). For the TUNEL assay wing discs from third instar larvae were dissected in PBS, fixed in 4% PFA in PBS and treated with the kit ApopTag Red (Chemicon) according to the manufacturer instructions. Images were taken using the Zeiss Apotome fluorescent microscope. For the *beclin1/atg6* mutant rescue experiment, third instar larvae were briefly rinsed in PBS and imaged on a Leica MZ16.5 stereoscope equipped with a digital camera.

Yeast Two-Hybrid screens

Yeast two-hybrid (Y2H) screening was performed by Hybrigenics Services (www.hybrigenics-services.com) with full-length human STK38 (Genbank: 31377778) and STK38-PIF [S7] as baits. cDNAs were cloned into pB27 (N-LexA-bait-C fusion) and used to screen a random-primed human fetal brain cDNA library constructed into pP6. More than 70 million clones (7-fold the complexity of the library) were screened with each bait using a mating approach previously described [S8]. Positive colonies were selected on a medium lacking tryptophan, leucine and histidine. Two clones coding for BCN1 were found in the screen with full-length STK38 and one clone in the screen with STK38-PIF.

Reagents, plasmids and siRNAs

Antibodies for Western blots were: LC3B (2775), Beclin1 (3495), cleaved-caspase 3 (9661), RalB (3523), p70(S6K) (2708), phospho-p70(S6K) (Thr389-P) (9206), phospho-ULK1 (Ser757-P) (6888) and Myc (2278) from Cell Signaling. SQSTM1 (PM045), β-Actin (A5441) with Flag (M2) and ULK1 (A7481), Adaptin (610502), GST (Ab6647) and GAPDH (MAB374) were from MBL, Sigma-Aldrich, Becton Dickinson (BD), Abcam and Millipore, respectively. Cleaved-PARP (asp214, 552597), STK38 (H00011329-M011), HA (11867423001) were from BD pharmingen, Abnova and Roche. Anti-phospho-STK38 (Thr444-P) and Mob1 antibodies were previously described [S9, S10]. Anti-HA 12CA5 antibody was used as hybridoma supernatant. For immunofluorescence studies: LC3B (5F10) and ATG12 (CAC-TMD-PH-ATG12) were from Nanotools and Cosmobio. BafilomycinA1, trehalose, tetracycline, 3-MA and EBSS were from Sigma-Aldrich and Gibco. Secondary antibodies donkey anti-mouse-HRP (115-035-003), donkey anti-rabbit-HRP (111-035-045) and donkey anti-rabbit light chain HRP (211-032-171) were from Jackson Immunoresearch and GE Healthcare. Plasmids for mRFP-GFP-LC3B (21074), pcDNA4-Beclin1-HA (24399), pEGFP-ATG14L (21635) and pDs-RED Beclin1 (24405) were from Addgene. STK38 [S9], Exo84-HA [S11], RalB [S12] plasmids were previously described. Vps34-RFP was a kind gift from Ktistakis NT (Babraham Institute, Cambridge, England, UK). siControl non-targeting siRNA (D-001810-01-50) was purchased from Dharmacon. The remaining siRNAs were purchased from Eurogentec or Qiagen:

STK38(1): 5’-CGUCGGCCAUAAACAGCUdTT-3’
STK38(2): 5’-GUAAUAGGCAGAGGAGCAdTT-3’
STK38(3): 5’-CCUUAUCGCUCAACAUGAAdTT-3’

RalA: 5’-GACAGGTTTCTGTAGAAGAdTT-3’
RalB(1): 5'-GAGCCCAGUAUUCACAUUUdTT-3'
RalB(2): 5’-UGACGAGUUUGUAGAAGACdTT-3’
Exo84: 5’-CCACTTTACTCTATATTCAdTT-3’
ULK1(1): 5’-CGCGGUACCUCCAGAGCAAdTT-3’
ULK1(2): 5’-CCCUUUGCGUUAUAUUGUAdTT-3’
The siRNAs targeting Beclin1 [S13] and MOB1A/B [S10] have been described.

Immunofluorescence, time lapse microscopy, and image analysis

For LC3B staining, cells were grown on glass coverslips, washed in PBS, fixed in 4% PFA for 10min, and subjected to cold-methanol treatment for 5min (-20°C). Aldehydes were quenched with 50mM NH_4Cl in PBS for 5min. After PBS washes, fixed cells were incubated in 0.1% Triton X-100 in PBS / 3% BSA for 10min, and then incubated for 30min with anti-LC3B antibody (2μg/mL). Cells were washed, before incubation with anti-mouse Alexa 488 secondary antibody for 30 min, followed by PBS and ddH2O washes and mounting in DAPI-containing mounting medium (Invitrogen). Images were acquired using a confocal microscope (A1R, Nikon). For quantifications, fields were chosen arbitrarily based on DAPI staining, and the number of LC3B dots per cell of at least 50 cells was determined with Image J software. For ATG12 staining, the cold-methanol treatment was skipped and images were acquired using an epifluorescence microscope (Eclipse, 90i, Nikon). Images for ATG14L and PI3P dots quantifications were acquired using an ApoTome 2 and evaluated with ImageJ. Co-localizations studies were carried out on a Leica TCS SP5 confocal microscope. For time lapse videos, images were acquired every 5 min using a spinning disk confocal microscope (Ti-E, Nikon).

Supplemental References

S1. Hergovich, A. (2013). Regulation and functions of mammalian LATS/NDR kinases: looking beyond canonical Hippo signalling. Cell Biosci. *3*, 32.
S2. Choi, J., Jung, W., and Koo, J. S. (2013). Expression of autophagy-related markers beclin-1, light chain 3A, light chain 3B and p62 according to the molecular subtype of breast cancer. Histopathology *62*, 275–286.
S3. White, E. (2012). Deconvoluting the context-dependent role for autophagy in cancer. Nat. Rev. Cancer *12*, 401–410.
S4. Qu, X., Yu, J., Bhagat, G., Furuya, N., Hibshoosh, H., Troxel, A., Rosen, J., Eskelinen, E.-L., Mizushima, N., Ohsumi, Y., et al. (2003). Promotion of tumorigenesis by heterozygous disruption of the beclin 1 autophagy gene. J. Clin. Invest. *112*, 1809–1820.
S5. Cornils, H., Stegert, M. R., Hergovich, A., Hynx, D., Schmitz, D., Dirnhofer, S., and Hemmings, B. A. (2010). Ablation of the kinase NDR1 predisposes mice to the development of T cell lymphoma. Sci. Signal. *3*, ra47.
S6. Mortensen, M., Watson, A. S., and Simon, A. K. (2011). Lack of autophagy in the hematopoietic system leads to loss of hematopoietic stem cell function and dysregulated myeloid proliferation. Autophagy *7*, 1069–1070.
S7. Cook, D., Hoa, L. Y., Gomez, V., Gomez, M., and Hergovich, A. (2014). Constitutively active NDR1-PIF kinase functions independent of MST1 and hMOB1 signalling. Cell. Signal. *26*, 1657–1667.

S8. Fromont-Racine, M., Rain, J. C., and Legrain, P. (1997). Toward a functional analysis of the yeast genome through exhaustive two-hybrid screens. Nat. Genet. *16*, 277–282.

S9. Hergovich, A., Bichsel, S. J., and Hemmings, B. A. (2005). Human NDR kinases are rapidly activated by MOB proteins through recruitment to the plasma membrane and phosphorylation. Mol. Cell. Biol. *25*, 8259–8272.

S10. Hergovich, A., Kohler, R. S., Schmitz, D., Vichalkovski, A., Cornils, H., and Hemmings, B. A. (2009). The MST1 and hMOB1 tumor suppressors control human centrosome duplication by regulating NDR kinase phosphorylation. Curr. Biol. CB *19*, 1692–1702.

S11. Parrini, M. C., Sadou-Dubourgnoux, A., Aoki, K., Kunida, K., Biondini, M., Hatzoglou, A., Poullet, P., Formstecher, E., Yeaman, C., Matsuda, M., et al. (2011). SH3BP1, an exocyst-associated RhoGAP, inactivates Rac1 at the front to drive cell motility. Mol. Cell *42*, 650–661.

S12. Cascone, I., Selimoglu, R., Ozdemir, C., Del Nery, E., Yeaman, C., White, M., and Camonis, J. (2008). Distinct roles of RalA and RalB in the progression of cytokinesis are supported by distinct RalGEFs. EMBO J. *27*, 2375–2387.

S13. Lefort, S., Joffre, C., Kieffer, Y., Givel, A.-M., Bourachot, B., Zago, G., Bieche, I., Dubois, T., Meseure, D., Vincent-Salomon, A., et al. (2014). Inhibition of autophagy as a new means of improving chemotherapy efficiency in high-LC3B triple-negative breast cancers. Autophagy *10*, 2122–2142.
